# An exceptionally stable octacobalt-cluster-based metal–organic framework for enhanced water oxidation catalysis†
†Electronic supplementary information (ESI) available: Experimental details, crystal data and structure refinement results, thermogravimetric analyses, variable-temperature PXRD. CCDC 1881071. For ESI and crystallographic data in CIF or other electronic format see DOI: 10.1039/c9sc03224j


**DOI:** 10.1039/c9sc03224j

**Published:** 2019-09-10

**Authors:** Ning-Yu Huang, Jian-Qiang Shen, Zi-Ming Ye, Wei-Xiong Zhang, Pei-Qin Liao, Xiao-Ming Chen

**Affiliations:** a MOE Key Laboratory of Bioinorganic and Synthetic Chemistry , School of Chemistry , Sun Yat-Sen University , Guangzhou 510275 , China . Email: liaopq3@mail.sysu.edu.cn; b Department of Chemical and Biomolecular Engineering , University of California , Los Angeles , CA 90095 , USA

## Abstract

Extensive efforts have been devoted to developing efficient and durable catalysts for water oxidation. Herein, we report a highly stable metal–organic framework that shows high catalytic activity and durability for electrically driven (an overpotential of 430 mV at 10 mA cm^–2^ in neutral aqueous solution) and photodriven (a turnover frequency of 16 s^–1^ and 12 000 cycles) water oxidation, representing the best catalyst for water oxidation reported to date. Computational simulation and isotope tracing experiments showed that the μ_4_-OH group of the {Co_8_(μ_4_-OH)_6_} unit participates in the water oxidation reaction to offer an oxygen vacancy site with near-optimal OH^–^ adsorption energy.

## Abstract 2

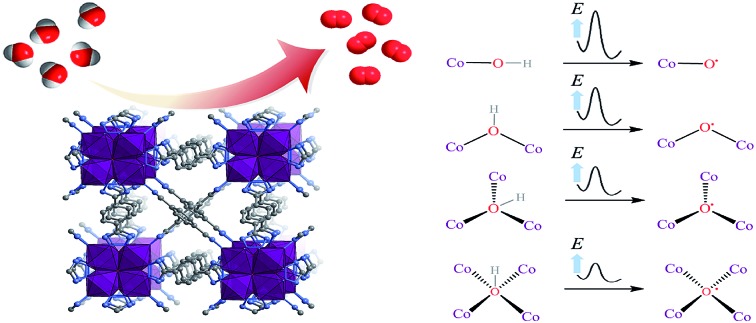
The site, capping four coplanar cobalt ions, has a near-optimal OH^–^ adsorption energy, which is beneficial to accelerate the reaction kinetics of water oxidation catalysis.

## Introduction

The replacement of fossil fuels with hydrogen generated by water splitting is a very attractive solution to the present energy problem. In order to develop a practical technology based on these elements, durable and efficient water oxidation catalysts need to be developed.^1^,^2^ However, the water oxidation process involves a four-electron process, leading to slow kinetics.^3^,^4^ For this reason, improving the efficiency of water oxidation catalysts is still a challenging task, despite the considerable achievements that have been made in recent years.^5^–^7^

**Scheme 1:**
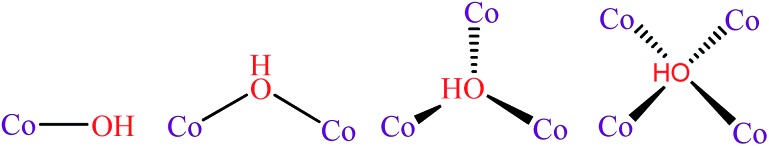
Proposed models of the reactant hydroxyl ion coordinated to the cobalt ions during the water oxidation reaction.

The active oxygen species (namely, the O˙ radical) has been accepted as the important intermediate for the formation of hydroperoxy (OOH) species and for the subsequent conversion to O_2_ molecules.^8^ Under alkaline conditions, the M–O˙ (M = metal) species is generated from the oxidation of the M–OH species, enhancing the adsorption energy of the reactant OH^–^, which might be beneficial to the formation of *OH for the oxygen evolution reaction (OER) intermediates. However, if OH^–^ binds too strongly, it will occupy available surface sites and poison the reaction.^9^ Therefore, optimizing the OH^–^ adsorption energy to a near-optimal value might be beneficial to accelerate the reaction kinetics.^10^ Obviously, regulating the coordination number of the oxygen atom of OH^–^ could be the most effective strategy to optimize the adsorption energy (Scheme 1). For instance, Jin *et al.* incorporated gold clusters onto the CoSe_2_ catalyst to increase the coordination number of the OH^–^ from 1 to 2, resulting in the enhancement of activity.^11^ Nevertheless, the binding affinity was still too weak, leading to a large amount of energy input required to produce *OH.

Metal–organic frameworks (MOFs), as crystalline porous materials with high surface areas and outstanding structural designability, can combine the advantages of both homogeneous and heterogeneous catalysts. Recently, MOFs have emerged as potential catalysts for water oxidation,^12^,^13^ the hydrogen evolution reaction,^14^–^17^ carbon dioxide reduction,^18^–^22^ *etc.*^14^,^23^–^25^ Nevertheless, as similar to traditional catalysts, the reported MOF catalysts also suffer from poor stability and low catalytic activity.^26^,^27^ Among the various types of MOFs, metal-azolate frameworks (MAFs) are famous for their extraordinary chemical stabilities.^28^ In addition, the high connectivity of the metal cluster could enhance the stability of the framework.^29^,^30^ Considering the relatively high activities and earth-abundance of cobalt ions, and that the multinuclear metal cluster might have a favourable OH^–^ adsorption energy, a combination of the highly connected cobalt-hydroxide unit and the azolate bridging ligand is the best choice. Here, we report a highly stable, octacobalt cluster based MAF with both extraordinarily high activity and durability. We demonstrated that the metal site, capping four coplanar cobalt ions, indeed serves as a highly efficient active site for water oxidation.

## Experimental section

### Materials and methods

All reagents were commercially available and used without further purification. 1,4-Benzenedi(1*H*-1,2,3-triazole (H_2_bdt) was synthesized according to the method in the literature. Elemental analyses (EA) were conducted using an Elementar Vario EL analyzer. X-ray photoelectron spectroscopy (XPS) measurements were performed with a VG Scientific ESCALAB 250 instrument. Powder X-ray diffraction (PXRD) patterns were collected on a Bruker D8-Advance diffractometer with Cu Kα radiation and a LynxEye detector. Variable-temperature PXRD data were collected on a Rigaku SmartLab X-ray diffractometer (Cu-Kα, *λ* = 1.54056 Å). Thermogravimetric (TG) analyses were performed on a TA Q50 thermogravimetric analyzer under nitrogen gas at a heating rate of 10 °C min^–1^. Scanning electron microscope (SEM) images were obtained from an ultra-high-resolution electron microscope (FE-SEM, SU8010). Gas sorption isotherms were measured on a Micromeritics ASAP 2020M instrument. Before the sorption experiments, the as-synthesized samples were first solvent exchanged with MeOH, and then activated for 12 h at 150 °C under vacuum. N_2_ (99.999%) was used for all measurements. The temperature was controlled by a liquid nitrogen bath (77 K).

### Synthesis of [Co_8_(OH)_4_(H_2_O)_2_(bdt)_6_]·guest (denoted as MAF-48 or **Co_4_-bdt**)

A mixture of Co(OAc)_2_·4H_2_O (17.5 mg, 0.75 mmol), H_2_bdt (10.5 mg, 0.5 mmol), triethylamine (TEA, 0.2 mL), H_2_O (1.0 mL) and *N*,*N*-diethylacetoacetamide (DEF, 4.0 mL) was stirred for 30 minutes in air, transferred to a 100 mL vial and sealed with a screw cap, heated in an oven at 160 °C for 72 h, and then cooled to room temperature at a rate of 10 °C h^–1^, giving red cubic crystals. The resulting red microcrystalline powders were washed with EtOH three times and then immersed in 1 M KOH (yield 78%). EA calc. (%) for [Co_8_(OH)_6_(bdt)_4_(Hbdt)_2_]·12H_2_O·5MeOH (C_65_H_88_N_36_Co_8_O_23_): C, 35.28; H, 4.01; N, 22.79; found: C 35.44, H 4.02, N 22.91.

### Crystal structure determination

Diffraction data of **Co_4_-bdt** were collected on a Rigaku XtaLAB P300DS-detector diffractometer (Cu Kα). All structures were solved by direct methods and refined with the full-matrix least-squares technique on *F*^2^ by the SHELXTL-2014 software package. All non-hydrogen atoms were refined anisotropically. Hydrogen atoms were placed geometrically. The PLATON SQUEEZE treatment was applied, because all guest solvent molecules were extremely disordered and could not be modeled. Detailed structure determination parameters and crystallographic data are given in Table S1.†

## Results and discussion

**Fig. 1:**
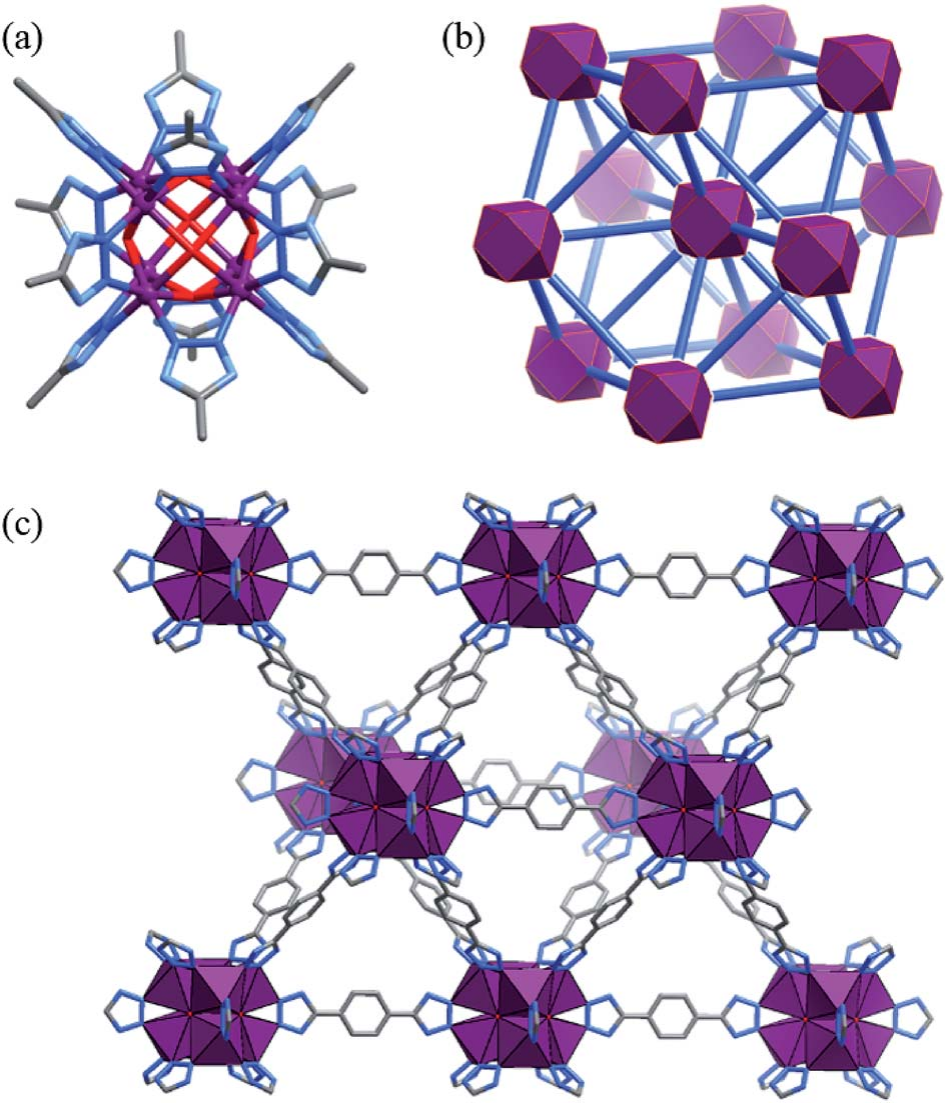
(a) The Co_8_(μ_4_-OH)_6_(Rtrz)_12_ cluster (hydrogen atoms are omitted for clarity), (b) the network topology (octanuclear clusters and bistriazolate ligands are simplified as violet polyhedra and blue sticks, respectively), and (c) 3D coordination framework (CoN_3_O_3_ units are shown as violet polyhedra) of **Co_4_-bdt**.

The solvothermal reaction of Co(OAc)_2_ and H_2_bdt in *N*,*N*-diethylformamide (DEF) afforded red cubic crystals of [Co_8_(OH)_6_(bdt)_4_(Hbdt)_2_] (MAF-48, **Co_4_-bdt**). Single-crystal X-ray analysis revealed that **Co_4_-bdt** consists of an **fcu** network constructed of 12-connected Co_8_(μ_4_-OH)_6_(Rtrz)_12_ (Rtrz^–^ = 1,2,3-triazolate group) clusters and 2-connected bdt^2–^ ligands (Fig. 1 and Table S1†), and this is isostructural with [Ni_8_(OH)_4_(H_2_O)_2_(bdp)_6_] (**Ni_4_-bdp**, H_2_bdp = 4,4′-benzene-1,4-diylbis(1*H*-pyrazole)).^31^ At each face of {Co_8_(μ_4_-OH)_6_}, the hydroxyl anion links four coplanar Co^II^ ions in a typical μ_4_ coordination mode to form a {Co_4_(μ_4_-OH)} unit, which is an underlying catalytic active site for the water oxidation reaction. X-ray photoelectron spectroscopy (XPS) of **Co_4_-bdt** showed that the metal ions are all divalent (Fig. S1†).

The purity of **Co_4_-bdt** was preliminarily demonstrated by the scanning electron microscopy (SEM) images and transmission electron microscopy (TEM) images (Fig. S2†). Thermogravimetric (TG) and powder X-ray diffraction (PXRD) measurements of methanol-exchanged **Co_4_-bdt** indicated that its guest molecules can be removed above 100 °C, and the host framework can be stable up to 300 °C (Fig. S3 and S4†). Moreover, **Co_4_-bdt** can remain intact in 6 M KOH (Fig. S5†) for at least 3 days, representing the most alkali stable MOF.^32^,^33^ A N_2_ sorption isotherm was measured for **Co_4_-bdt** at 77 K (Fig. S6†), which shows typical type-I characteristics, with a saturation uptake of 594 cm^3^ (STP) g^–1^, corresponding to a pore volume of 0.928 cm^3^ g^–1^ (crystallographic value of 0.924 cm^3^ g^–1^). Furthermore, the Brunauer–Emmett–Teller (BET) and Langmuir surface areas of **Co_4_-bdt** were calculated to be 2266 and 2570 m^2^ g^–1^, respectively.

**Fig. 2:**
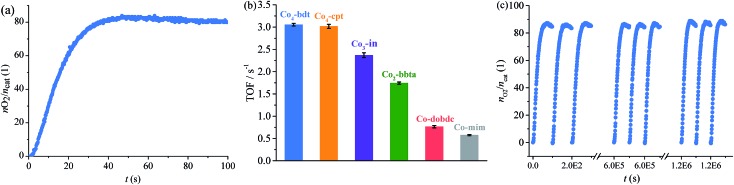
(a) Kinetics of O_2_ formation in the photocatalytic system using **Co_4_-bdt** as the catalyst. (b) Comparison of the TOF values for the photodriven water oxidation reactions. (c) O_2_ production profiles of the repeated photocatalytic water oxidation reactions using **Co_4_-bdt** as the catalyst. Reaction conditions: [Ru(bpy)_3_]SO_4_ (0.03 μmol), catalyst (0.5 nmol), borate buffer (pH = 9, 2 mL), Na_2_S_2_O_8_ (0.1 μmol), LED light (*λ* = 450 ± 5 nm), and 25 °C.

The photodriven water oxidation (PWO) experiment of **Co_4_-bdt** was performed under visible light in water with [Ru(bpy)_3_]SO_4_ (bpy = 2,2′-bipyridine) as the photosensitizer and Na_2_S_2_O_8_ as the sacrificial electron acceptor, and these are the typical reaction conditions used in the literature. A Clark-type oxygen electrode was used to monitor *in situ* the amount of evolved O_2_ dissolved in the solution (Fig. 2a and S7†). O_2_ rapidly formed at an initial turnover frequency (TOF) of 3.05 ± 0.03 s^–1^. Due to the consumption of the sacrificial electron acceptor, the production rates slowly decreased (Fig. S8†). Except for PSII, the performance of **Co_4_-bdt** is higher than that for all other known heterogeneous catalysts,^26^,^27^,^34^–^39^ and is comparable to the best homogeneous catalysts under the same conditions^40^–^43^ (Tables S2 and S3†). Since the process that limits catalytic turnover is the oxidative quenching of the Ru excited state,^44^–^46^ the photocatalytic water oxidation experiments using [Ru(bpy)_3_]^3+^ as the chemical oxidant without Na_2_S_2_O_8_ were carried out. It can be seen that the TOF values increased and that the catalysts work under pseudo first order conditions (Fig. S9†). Importantly, the actual TOF value of **Co_4_-bdt** is as high as 15.7 s^–1^ (Fig. S10†), which is higher than that of the best PWO catalyst (13 s^–1^).^47^–^50^

Thanks to the excellent stability, the TOF of **Co_4_-bdt** remained 3.05 s^–1^ (Fig. 2c) after 12 000 runs, indicating that the turnover number (TON) is larger than one million. Notably, the TON value of **Co_4_-bdt** is two orders higher than that of the best catalysts.^51^ To directly determine the TON of the **Co_4_-bdt**, time-dependent oxygen evolution experiments were carried out with chemical oxidant [Ru(bpy)_3_]^3+^. As shown in Fig. S11,† **Co_4_-bdt** gave a TON of 1.2 × 10^6^, which is consistent with that obtained from the recycling experiment (Fig. 2c). After the reactions, the **Co_4_-bdt** catalyst was recovered from the reaction mixture and was found to be almost unchanged according to the N_2_ sorption isotherm measurements (Fig. S6†), the PXRD patterns and the SEM images (Fig. S12†). Furthermore, inductively coupled plasma-mass spectrometry showed that just 0.14% of the Co ions in **Co_4_-bdt** were leached into the reaction solution after the reaction, and the filtrate showed negligible catalytic activity (Fig. S7†), which confirmed the heterogeneous nature and the stability of the catalyst. The high stability of **Co_4_-bdt** might be ascribed to the high connectivity of the second building units (SBUs)^29^ and the more stable cobalt–N (nitrogen atom) coordination bonds.^28^,^32^

In order to study the mechanism, we selected five other cobalt-based MOFs with variable metal-hydroxide units and an isostructural MOF, [Ni_8_(OH)_6_(bdt)_4_(Hbdt)_2_] (**Ni_4_-bdt**), for comparison (Fig. S13–S15†): (i) [CoII8(μ_4_-OH)_6_(cpt)_6_] (**Co_4_-cpt**, Hcpt = 4-(4′-carboxyphenyl)-1,2,4-triazole) is made up of octanuclear cobalt-hydroxide {Co_8_(μ_4_-OH)_6_} clusters and cpt^–^ ligands;^52^ (ii) [Co_6_(μ_3_-OH)_2_(in)_4_(HCOO)_6_] (**Co_3_-in**, Hin = isonicotinic acid) is made up of asymmetric triangular units of {Co_3_(μ_3_-OH)}, in^–^ and oxalate ligands.^53^ Each hydroxyl anion is linked to three adjacent Co^II^ ions in a typical μ_3_ coordination mode to build the {Co_3_(μ_3_-OH)} cluster; (iii) [Co_2_(μ-OH)_2_(bbta)] (MAF-X27-OH/**Co_2_-bbta**, H_2_bbta = 1*H*,5*H*-benzo-(1,2-*d*:4,5-*d*′)bistriazole) bears a pair of μ-OH^–^ ligands at the *cis*-positions of its open metal site.^32^ Each hydroxyl anion links two adjacent Co^II^ ions in a typical bidentate coordination mode to build a {Co_2_(μ-OH)} unit; (iv) [Co_2_(dobdc)] (Co-MOF-74/**Co-dobdc**, H_4_dobdc = 2,5-dihydroxyl-1,4-benzenedicarboxylic acid) is composed of square-pyramidal Co ions and dobdc^4–^ ligands.^54^ The square-pyramidal Co ion can coordinate to a terminal water molecule or to a hydroxyl anion to form a distorted octahedral mode; (v) [Co(mim)_2_] (ZIF-67/**Co-mim**, Hmim = 2-methylimidazole) is constructed of tetrahedral Co ions and mim^–^ ligands.^55^ In addition, the tetrahedral Co ion can coordinate to a terminal water molecule or to a hydroxyl anion to form a distorted trigonal-bipyramidal mode.

The PWO experiment was performed under the same conditions. As calculated from the initial O_2_ production rates (Fig. 2b and S16†), the turnover frequency (TOF) for O_2_ is as follows, **Co_4_-bdt** (3.05 ± 0.03 s^–1^) ≈ **Co_4_-cpt** (3.02 ± 0.05 s^–1^) > **Co_3_-in** (2.37 ± 0.05 s^–1^) > **Co_2_-bbta** (1.74 ± 0.03 s^–1^) > **Ni_4_-bdt** (1.21 ± 0.03 s^–1^) > **Co-dobdc** (0.77 ± 0.03 s^–1^) ≈ **Co-mim** (0.58 ± 0.01 s^–1^) (Tables S4 and S5†). Interestingly, for the cobalt ions, when the coordination number of the hydroxide ligand increases, the catalytic performance becomes better, and the catalytic performance of **Co_4_-bdt** is much higher than that of **Ni_4_-bdt**.

**Fig. 3:**
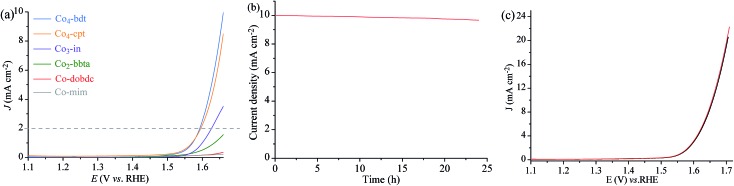
(a) LSV curves of **Co_4_-bdt**, **Co_4_-cpt**, **Co_3_-in**, **Co_2_-bbta**, **Co-mim** and **Co-dobdc** at pH = 7. (b) The chronopotentiometry curves of **Co_4_-bdt** at an overpotential corresponding to the current density of 10 mA cm^–2^ at pH = 7. (c) LSV curves of **Co_4_-bdt** before (black) and after (red) the electrochemical OER test at 10 mA cm^–2^ for 24 h at pH = 7.

To demonstrate that the Co-manifold works with a four electron/four proton mechanism, the activity of **Co_4_-bdt** for water oxidation was studied by electrochemical characterization. Linear sweep voltammetry (LSV) was performed in water at pH = 7 (Fig. 3a, S17 and S18†). Assuming that the water oxidation reaction involves a four-electron process, the Faraday efficiency of **Co_4_-bdt** for water oxidation was measured to be virtually 100% (Fig. S19 and Table S6†). Importantly, **Co_4_-bdt** afforded a current density of 2.0 mA cm^–2^ at an overpotential of 352 mV, which is much lower than that for all other reported catalysts (Table S7†). The performance of **Co_4_-bdt** showed negligible changes after OER tests at 10 mA cm^–2^ for 24 h (Fig. 3b). Furthermore, PXRD patterns (Fig. S12†) and cyclic voltammetry curves (Fig. 3c) of **Co_4_-bdt** showed negligible changes after the electrochemical OER tests for 24 h. The electrocatalytic activity follows the order: **Co_4_-bdt** (352 mV) > **Co_4_-cpt** (355 mV) > **Co_3_-in** (385 mV) > **Co_2_-bbta** (489 mV) > **Co-dobdc** (544 mV) > **Co-mim** (638 mV) (Fig. S17a and Table S7†) and this is consistent with results observed from the photodriven water oxidation experiment. This phenomenon demonstrates the high catalytic activity of **Co_4_-bdt** in the water oxidation reaction.

**Fig. 4:**
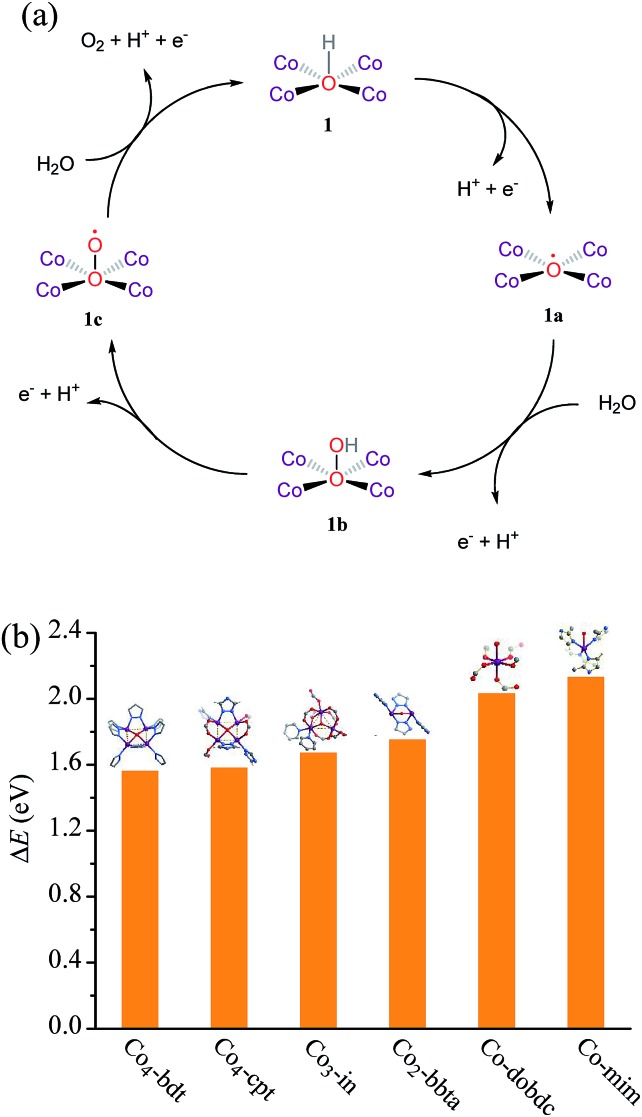
(a) A proposed reaction mechanism for using **Co_4_-bdt** as the catalyst for photodriven water oxidation. (1) The complex **1** is oxidatively activated by the photo-generated hole. (2) The nucleophilic attack of the water molecule forms the O–O bond. (3) The complex **1b** is oxidized to complex **1c**. (4) The complex **1c** is further oxidized to evolve O_2_, accompanied by the regeneration of **1**. (b) PDFT calculated adsorption energies of the reacting hydroxyl radical (Δ*E* = *E*(*OH) – *E*(*) – [*E*(H_2_O) – *E*(H)], * represents the catalyst).

Isotope tracing experiments were carried out to investigate the role of μ_4_-OH^–^ during the water oxidation reaction. The extent of ^18^O catalyst incorporation was calculated to be *ca.* 44.8 ± 1.1%, measured by the GC-MS analysis of the acidolyzed sample (Fig. S20 and Table S8†). During a representative water oxidation experiment with ^18^O labeled **Co_4_-bdt** as the photocatalyst, the photogenerated ^18^O^16^O (*m*/*z* = 34) could be clearly detected, while ^18^O^16^O was not detected in the water oxidation experiment with unlabeled **Co_4_-bdt** (Fig. S21, S22 and Table S9†). After the ^18^O-labeled **Co_4_-bdt** was immersed in pH = 9 aqueous solution H_2_^16^O for 10 min, ^18/16^O_2_ intensity was the same as that of the fresh ^18^O-labeled sample (Fig. S23†). This demonstrates that the ^16^O/^18^O exchange behaviour between the water molecule and the cobalt-hydroxide {Co_4_(μ_4_-OH)} unit during the isotope tracing experiment can be neglected for the observed significant ^18/16^O_2_ intensity enhancement. This result indicates that the bridging OH^–^ ligand does indeed participate in the reaction to offer an oxygen vacancy, which serves as the active site for water oxidation. This phenomenon was also observed for **Co_3_-in** and **Co_2_-bbta** (Fig. S21†). In other words, the site, capping four coplanar cobalt ions, indeed serves as the highly efficient active site for water oxidation. Such an active site for water oxidation is the first to be reported to date. It should be noted that not all oxygen atoms from the Co_4_ cluster participate in the reaction at the same time, and the O coordinated on the Co_4_ cluster will be replaced by the water molecule before the O_2_ formation. Therefore, although the O from the Co_4_ cluster indeed engages in O_2_ formation, the structure of **Co_4_-bdt** remains during and after the photodriven water oxidation reaction (Fig. 4a, S12 and S24†).

To further understand the relationship between the coordination number of the oxygen atom in OH^–^ and the properties, we analyzed the adsorption energy of OH^–^ adsorbed on the active site by periodic density functional theory (PDFT). The adsorption energy (Δ*E*) of OH^–^ follows the order, **Co-mim** (2.13 eV) > **Co-dobdc** (2.03 eV) > **Co_2_-bbta** (1.75 eV) > **Co_3_-in** (1.67 eV) > **Co_4_-cpt** (1.58 eV) ≈ **Co_4_-bdt** (1.56 eV) > **Ni_4_-bdt** (1.19 eV) (Fig. 4b), and this implies that the OH^–^ becomes more stable with an increase in the coordination number. The poor performance of **Ni_4_-bdt** is due to its too strongly OH^–^ binding. By combining the PDFT simulation results and the isotope tracing experiments, it can be seen that the high catalytic performance of **Co_4_-bdt** might be ascribed to the fact that the OH^–^ is appropriately stabilized by four coplanar cobalt ions.

## Conclusions

In conclusion, through a combination of the highly connected cobalt-hydroxide unit and the azolate bridging ligand, we designed and synthesized cobalt azolate frameworks with high stability and excellent water oxidation performance. Since the oxygen atom is simultaneously coordinated by four coplanar cobalt ions, the reacting hydroxyl radical is appropriately stabilized during the water oxidation reaction, promoting the catalytic performance. These results should be insightful for understanding the structure–function relationship of water oxidation catalysts and for developing new MOF-based catalysts.

## Conflicts of interest

There are no conflicts to declare.

## Supplementary Material

Supplementary information

Crystal structure data
