# Supplementary material for: An exceptionally stable octacobalt-cluster-based metal–organic framework for enhanced water oxidation catalysis
Source: Chem Sci. 2019 Sep 10;10(42):9859–64. doi: 10.1039/c9sc03224j (PMC6977550; doi:10.1039/c9sc03224j)
Supplement: Supplementary file 1 [file SC-010-C9SC03224J-s001.pdf]

Electronic Supplementary Material (ESI) for Chemical Science.
This journal is © The Royal Society of Chemistry 2019

Electronic Supplementary Information (ESI) for

An Exceptionally Stable Octacobalt-Cluster-Based Metal-Organic Framework for Enhanced Water Oxidation Catalysis

Ning-Yu Huang,[1#] Jian-Qiang Shen,[1,2#] Zi-Ming Ye,[1] Wei-Xiong Zhang,[1] Pei-Qin Liao,[1*] and Xiao-Ming Chen[1]

[1] *MOE Key Laboratory of Bioinorganic and Synthetic Chemistry, School of Chemistry, Sun Yat-Sen University, Guangzhou 510275, China*

[2] *Department of Chemical and Biomolecular Engineering, University of California Los Angeles, CA 90095, USA*

[#] *These authors contributed equally to this work.*

***E-mail:** **liaopq3@mail.sysu.edu.cn**

Supplementary Index

Experimental details.

Figure S1. XPS spectra of **Co_4-bdt**.

Figure S2. SEM, TEM images and the corresponding particle size distribution of **Co_4-bdt**.

Figure S3. PXRD patterns of **Co_4-bdt** after activated and variable-temperature PXRD of **Co_4-bdt**.

Figure S4. TG curves of **Co_4-bdt**.

Figure S5. PXRD patterns of **Co_4-bdt** after immersed in 6 M KOH.

Figure S6. N_2 adsorption and desorption isotherms of **Co_4-bdt** at 77 K.

Figure S7. Control photocatalytic experiments using **Co_4-bdt** as catalyst.

Figure S8. Different approaches to regenerate the PWO activity of **Co_4-bdt**.

Figure S9. Dependence of initial rate of O_2 production on **Co_4-bdt** and other MOFs.

Figure S10. Kinetics of O_2 formation in the oxidation reaction with chemical oxidants $[Ru(bpy)_3]^{3+}$.

Figure S11. Time-dependent oxygen evolution curves of **Co_4-bdt**.

Figure S12. Stability tests of **Co_4-bdt** after photocatalytic measurements.

Figure S13. Framework structures and coordination geometries of metal centers of the catalysts.

Figure S14. PXRD patterns of the catalysts.

Figure S15. SEM images of the catalysts

Figure S16. O_2 production profiles of the repeated photocatalytic water oxidation reactions.

Figure S17. Cyclic voltammetry curves for **Co_4-bdt** at pH = 7 and the capacitive current density (Ja/2-Jc/2) as a function of scan rate in the region of 0.85~1.25 V *vs*. RHE.

Figure S18. Onset potentials and Tafel plots of **Co_4-bdt** and other MOFs.

Figure S19. Gas chromatography profiles of O_2 and N_2 in the electrolytic cell using **Co_4-bdt** as anode after 0 and 9000 s electrolysis.

Figure S20. Dependence of the peak ratio of $H_2{}^{18}O/H_2{}^{16}O$ and GC-MS profiles of the acidolyzed **Co_4-bdt**.

Figure S21. GC-MS profile of PWO experiments with ^{18}O-labeled or unlabeled catalysts.

Figure S22. GC-MS profiles of the repeated photocatalytic water oxidation reactions.

Figure S23. GC-MS profiles of PWO experiments with ^{18}O-labeled **Co_4-bdt** (in pH=9 aqueous solution ($H_2{}^{16}O$) for 10 min) and ^{18}O-labeled **Co_4-bdt** (after PWO experiment).

Figure S24. Rietveld refinement plots of **Co_4-bdt** before and during PWO experiment.

Table S1. Crystallographic Data for **Co_4-bdt**.

Table S2. Comparison of the photocatalytic activities of **Co_4-bdt** and heterogeneous catalysts.

Table S3. Comparison of the photocatalytic activities of **Co_4-bdt** and homogeneous catalysts.

Table S4. Summary of the results of the photocatalytic water oxidation.

Table S5. Summary of the results of the repeated photodriven water oxidation reaction.

Table S6. The obtained gas chromatography peak areas of O_2/N_2 in the electrolytic cell.

Table S7. Comparison of the OER activities of **Co_4-bdt** and benchmarked catalysts working at pH = 7.

Table S8. Summary of the $H_2{}^{16}O$ and $H_2{}^{18}O$ peak intensity of the acidolyzed **Co_4-bdt**.

Table S9. Summary of the $^{18/16}O_2$ peak intensity of the photodriven water oxidation reaction.

Experimental section

Photocatalytic Measurements. A solution of 10 mL borate buffer containing (10 mg, 5 μmol) **Co_4-bdt** was prepared and then 0.1 mL of the as-prepared solution was dispersed in 9.9 mL fresh borate buffer. After the borate buffer solution of **Co_4-bdt** was mixed with $[Ru(bpy)_3]SO_4$ and $Na_2S_2O_8$, the reaction mixture was purged with argon for 5 min and then the reaction was initiated with light irradiation. The total volume of the solution was 2.0 mL. The generation of dissolved oxygen was analyzed by a Hanstech Clark-type electrode irradiated under an LED light ($\lambda = 450 \pm 5$ nm, with a light intensity of 100 mW cm^{-2} and irradiated area of approximately 0.5 cm^2). Each photocatalytic reaction was repeated three times to confirm the reliability of the data. The turnover frequency (TOF) was calculated using the initial rates of gas evolution (the slope of $n_{O2}/n_{catalyst}$ – time curves).

The concentration of Co in the filtrate was detected by the inductively coupled plasma–mass spectrometry. After PWO reaction (500 nmol catalysts in 2 mL solution), 1 mL of the filtrate was used for tests. The concentration of Co in the concentrated filtrate was detected as 6.05 ng/mL. Therefore, the dissolved Co ions of the MOF was 6.05 ng mL^{-1} × 25 mL)/(250 nmol × 8 × 59 g mol^{-1}) = 0.13%).

The determination of the total TON was performed as described below. A borate buffer solution (50 mM, pH 9.0, 50 mL) containing $Na_2S_2O_8$ (20.0 mM) and a borate buffer solution (50 mM, pH 9.0, 50 mL) containing $[Ru(bpy)_3]SO_4$ (10.0 mM) were prepared and syringe. Because the photosensitizer and sacrificial agents is easy to be deactivated under irradiation[1], two syringes carrying $Na_2S_2O_8$ and $[Ru(bpy)_3]SO_4$ solution respectively were placed at the dual channel syringe pump separately and both solutions were slowly injected into the quartz reactor at the rate of 5 μL/min. During the injection, the solution was irradiated under an LED light. After one week or two weeks, the catalysts were recovered from the solution and used for the PWO reaction immediately.

Considering the time of PWO reaction is 100 s for each run, 1 or 2 weeks (604800 s or 1209600 s) of the injection and irradiation can be regarded as 6048 or 12096 rounds of recycle tests. As the performance of **Co_4-bdt** does not decline after two weeks, the total TON can be calculated as 12096 times of the TON in a single run, which is 83 determined by a single PWO run. Therefore, the total TON of **Co_4-bdt** > 12096 * 83 = 1003968

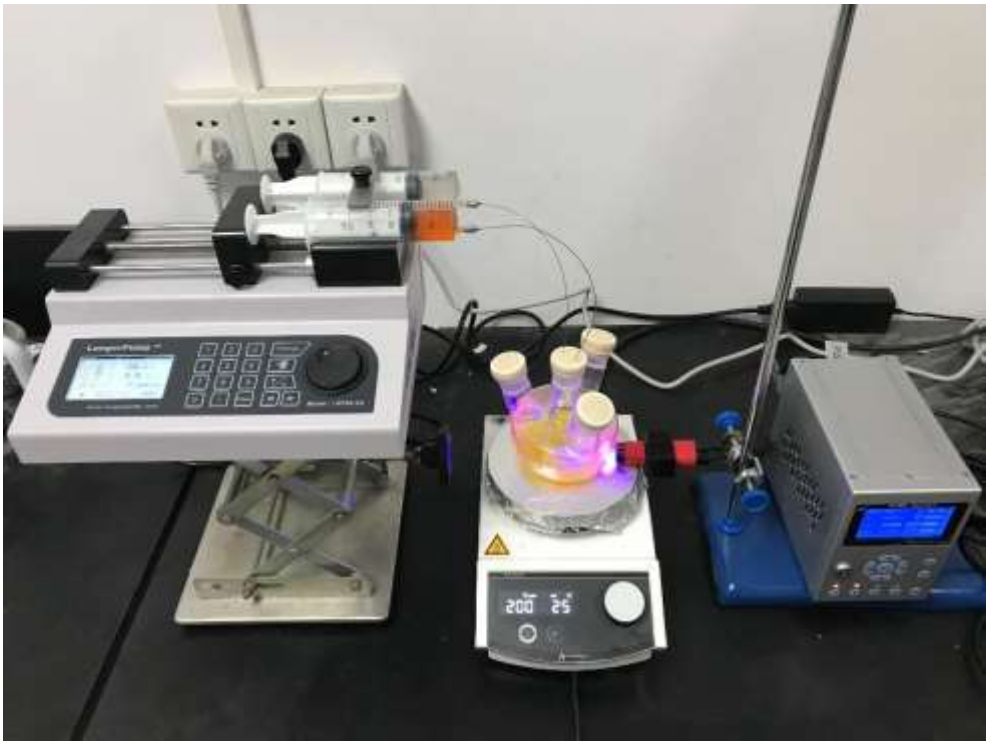

Scheme S1 Photo of the photodriven water oxidation recycle system

Isotope labelling experiment. Isotopically pure ^{18}O-labeled **Co4-bdt** can be prepared via hydrothermal reaction with heavy-oxygen water, $H_2{}^{18}O$. ^{18}O-labeled **Co3-in** was synthesized via solvothermal reaction with $H_2{}^{18}O$ as solution. ^{18}O-labeled **Co2-bbta** was synthesized according to the previous literature,[2] except that the $H_2{}^{16}O$ solution of KOH was replaced by a $H_2{}^{18}O$ solution of KOH. Isotopic tracing experiments (ITEs) were carried out by using ^{18}O-labeled or unlabeled **Co4-bdt, Co3-in** and **Co2-bbta** as photocatalyst. The suspensions were prepared by mixing 5 mL of the solvent ($H_2{}^{16}O$) with 10 mg of the catalyst (^{18}O-labeled or unlabeled catalysts), 77 mg of $[Ru(bpy)_3]SO_4$ and 120 mg of $Na_2S_2O_8$. Before illumination, the reactor was purged with N_2 gas (flow = 30 ml min^{-1}) for 20 minutes in order to remove dissolved oxygen. The gas in the headspace was withdrawn using a gas-tight syringe and analyzed by GC-MS as the background. Then the light was turned on for 10 minutes and gas in the headspace was withdrawn and analyzed as the sample signal. The intensity of each signal from mass spectrum was calculated with background correction.

Preparation of working electrodes. Co4-bdt microcrystals coated on GCE using Nafion as binder: A suspension was prepared by dispersing **Co4-bdt** microcrystals (10 mg) in a mixed solution

(2 mL) containing ethanol (1.9 mL) and a 5 wt% Dupont Nafion 117 solution (0.1 mL), followed by ultrasonication for 30 min. Then the suspension (8 μL) was pipetted on a GCE surface of a rotating disk electrode (RDE, 5 mm in diameter, Pine Research Instrumentation). Prior to pipette, the GCE was polished carefully with 1.0, 0.3 and 0.05 μm alumina powder, successively, and rinsed with ultrapure water, followed by ultrasonication in ethanol and ultrapure water successively. The working electrode was dried at ambient temperature before electrochemical measurements.

Electrochemical Measurements. All measurements were performed in an RDE setup equipped with a three compartment electrochemical glass cell, a Luggin capillary, a PINE rotator (Pine Research Instrumentation), and a CHI 760E electrochemical workstation at room temperature with the O_2 saturated electrolyte, which was a 1.0 M potassium phosphate buffer (pH = 7) for OER. LSV was performed at a scan rate of 5 mV s^{-1} and an electrode spin rate of 1200 rpm. All potentials were referenced to a saturated calomel electrode (SCE) reference electrode, and Pt wire was used as the counter electrode in all measurements. All potentials were adjusted to compensate for the ohmic potential drop losses (R_s) that arose from the solution resistance and calibrated with respect to reversible hydrogen electrode (RHE). $E_{\text{vs.RHE}} = E_{\text{vs.SCE}} + 0.2412 + 0.05916\text{pH-iR}_s$.

Measurement of the Faraday efficiency. A piece of glass electrode (surface area = 0.5 cm^2) attached with microcrystalline **Co_4-bdt**, was used as the working electrode. The head space volume of the electrolytic cell and the volume of solution were measured to be 32.0 and 28.0 mL, respectively. Before measurement, the cell was purged by Ar gas for about 30 min to reduce the O_2 background. The oxygen concentrations were measured using gas chromatography (Agilent 7820A) equipped with Molecular sieve 5 Å capillary column and thermal conductivity detector.

A linear relationship between the O_2 concentration and its GC peak area was obtained by plotting three GC measurements of calibrated diluted O_2 samples:

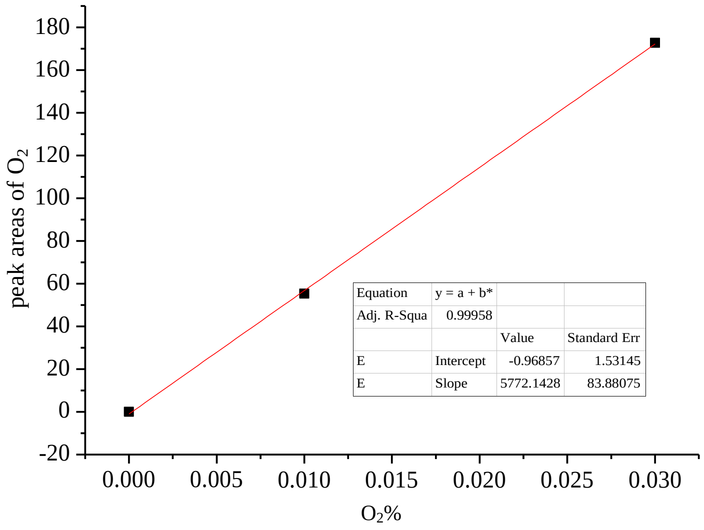

The turnover frequency (TOF) of the catalyst for OER is defined as

TOF = $n_{O2}/n_{cat}/t$

where n_{O2} is the amount of oxygen (mol) produced, n_{cat} is the amount of catalytic active centers in the catalyst (mol) and t is the electrolysis time (s).

When the electrolysis current is all used for OER,

$TOF_{theoretical} = J/(4 \times F \times m/M)$

Where J is the current density (mA cm^{-2}) at a given overpotential, F is the faraday constant (96485 C mol^{-1}), m is the mass loading of the catalyst (mg cm^{-2}), and M is the molecular weight of the catalyst unified with one active center per formula unit.

The Faraday efficiency for OER is calculated by

$F_{OER} = TOF / TOF_{theoretical} \times 100\%$

Computational Methods. All simulations/calculations were performed by the Materials Studio 5.0 package. All energies were calculated by the periodic density functional theory (PDFT) method by the Dmol3 module. Full geometry optimizations with fixed cell parameters were performed to the systems. The widely used generalized gradient approximation (GGA) with the Perdew-Burke-Ernzerhof (PBE) functional and the double numerical plus d-functions (DNP) basis set, TS for DFT-D correction as well as the Effective Core Potentials (ECP) were used. The energy, force and displacement convergence criterions were set as 1×10^{-5} Ha, 2×10^{-3} Ha and 5×10^{-3} Å, respectively.

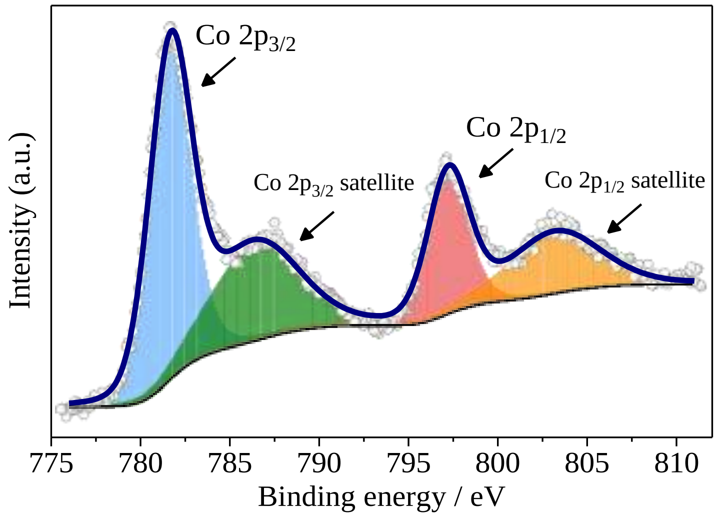

Figure S1. XPS spectrum of **Co$_4$-bdt**. For **Co$_4$-bdt**, the Co 2p$_{3/2}$ and 2p$_{1/2}$ peaks appear at *ca*. 781.7 eV and 797.2 eV and the Co 2p$_{3/2}$ and 2p$_{1/2}$ satellite peaks appear at *ca*. 786.4 eV and 803.2 eV, indicating the presence of Co(II) instead of Co(III).

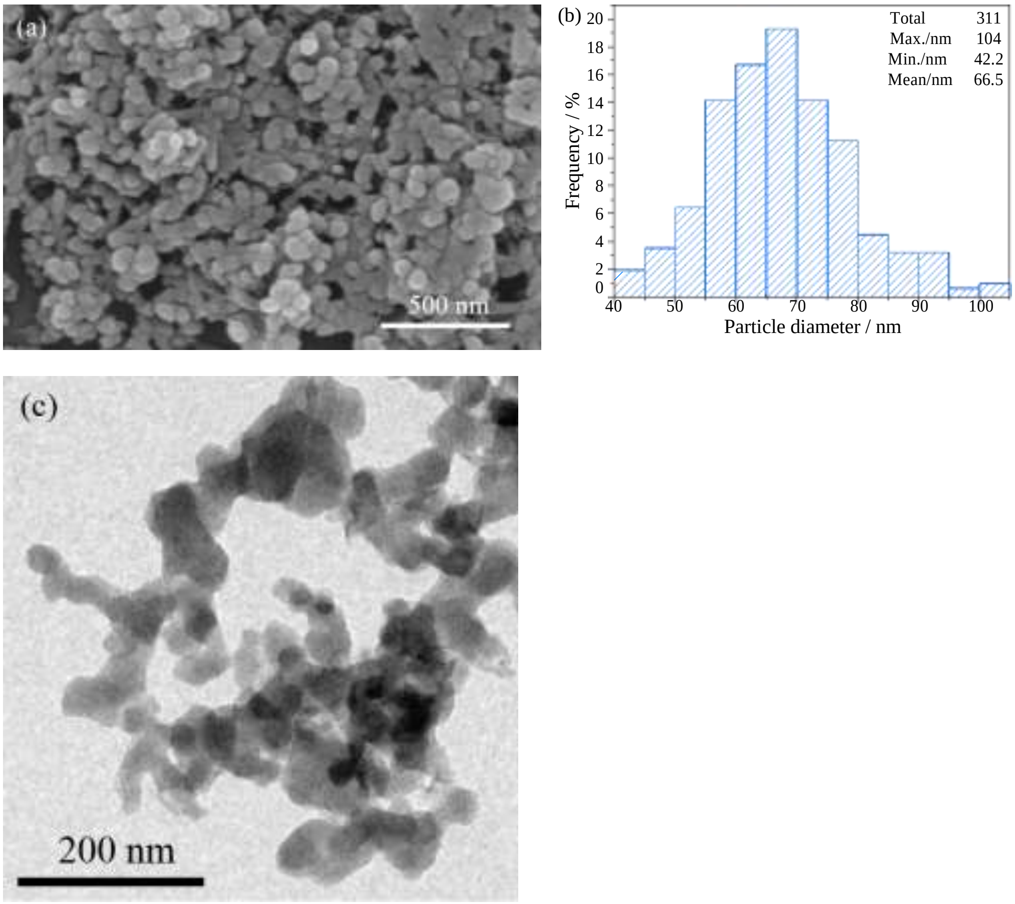

Figure S2. (a) SEM images and (b) the corresponding particle size distribution of **Co$_4$-bdt**. (c) TEM images.

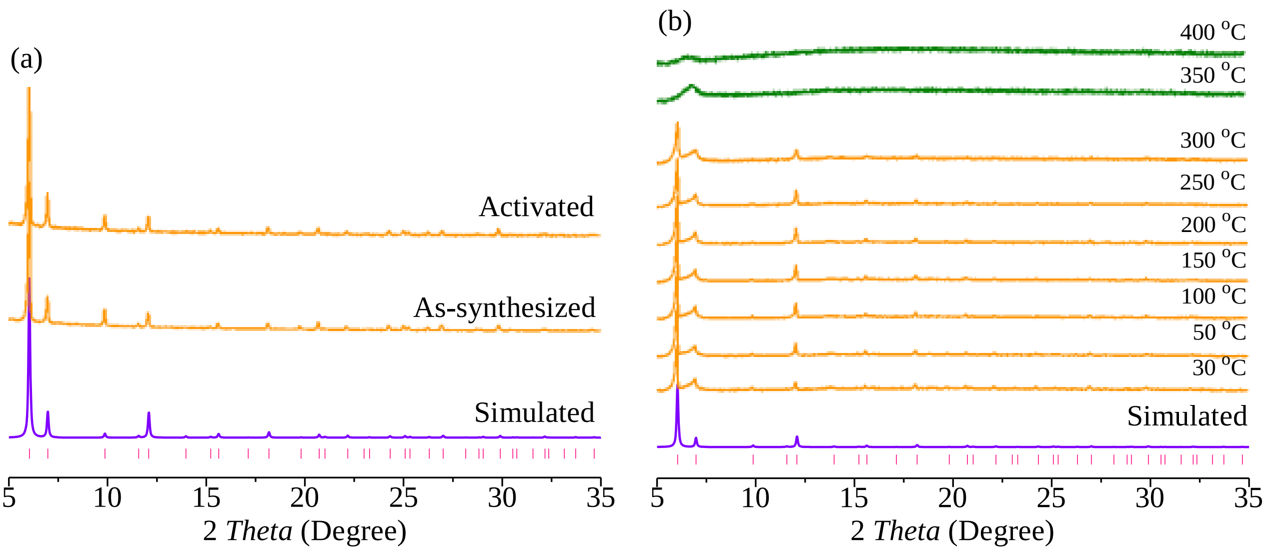

Figure S3. (a) PXRD patterns of **Co_4-bdt** after activated at 150 °C under N_2 for 30 min and (b) after heated at different temperatures under N_2 for 30 min.

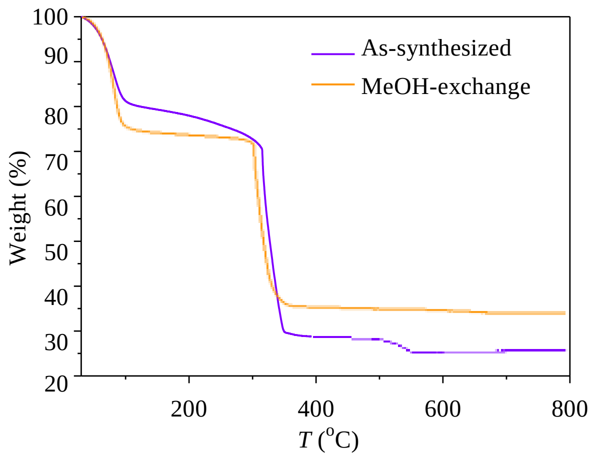

Figure S4. TG curves of **Co_4-bdt**. For the as-synthesized sample, there is a small weight decline between 500 and 600 °C in the TGA curve, which might be ascribed to amorphous impurity. The amorphous impurity could be removed by the treatment of MeOH-exchanged.

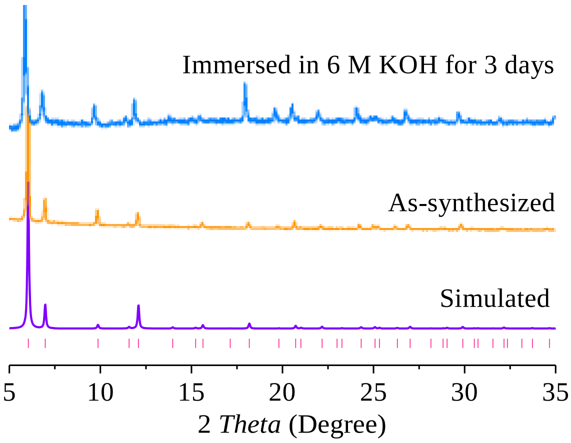

Figure S5. PXRD patterns of **Co4-bdt** after immersed in 6 M KOH.

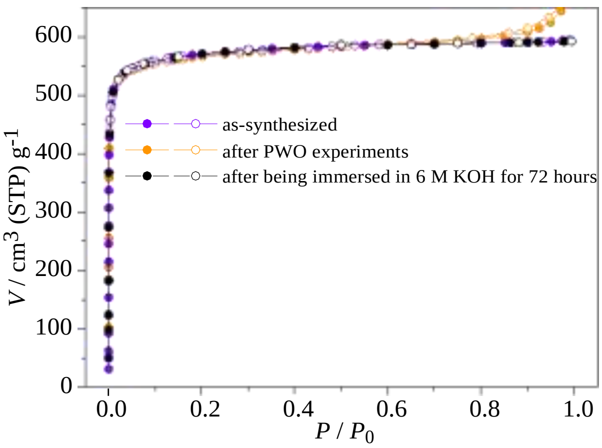

Figure S6. N_2 adsorption (solid) and desorption (open) isotherms of **Co4-bdt** at 77 K.

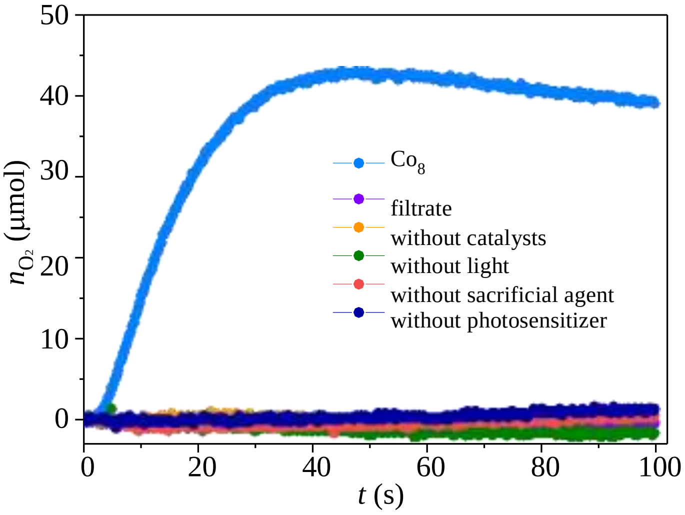

Figure S7. Control photocatalytic experiments using **Co_4-bdt** as catalyst.

We further carried out a series of control experiments for **Co_4-bdt** (Figure S13). When the reaction was operated in the absence of the MOF, light irradiation, photosensitizer, or sacrificial agent, just trace amount of O_2 were detected, demonstrating the catalysis role of the MOF and necessities of the photosensitizer, sacrificial agent, and visible-light.

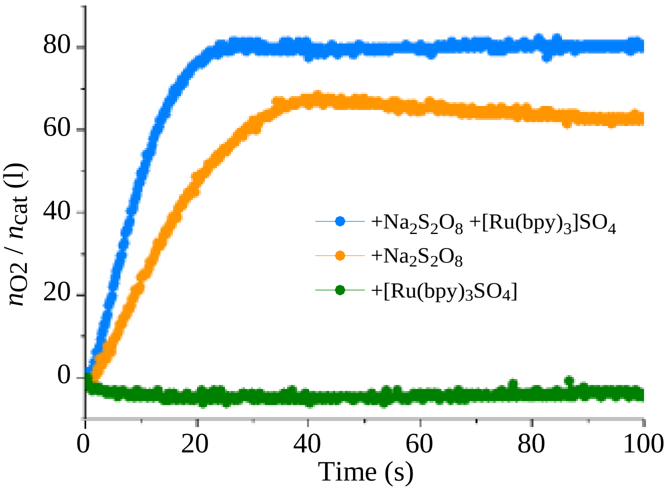

Figure S8. Using **Co_4-bdt** as catalyst, addition of $Na_2S_2O_8$ + $[Ru(bpy)_3]^{2+}$ (blue line), $Na_2S_2O_8$ (orange line) and $[Ru(bpy)_3]^{2+}$ (green line), respectively, to regenerate the PWO activity.

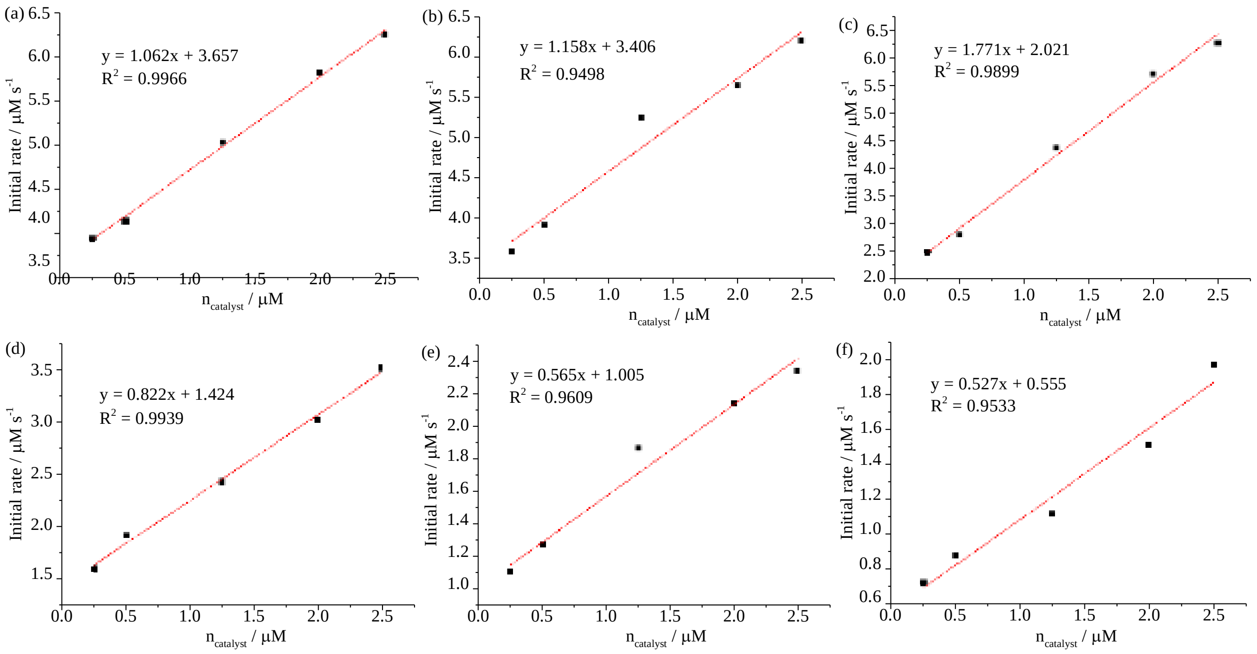

Figure S9. Dependence of initial rate of O_2 production on (a) **Co_4-bdt** (b) **Co_4-cpt**; (c) **Co_3-in**; (d) **Co_2-bbta**; (e) **Co-mim** and (f) **Co-dobdc** concentration at 15 μM $[Ru(bpy)_3]^{3+}$. Conditions: 80 mM sodium borate buffer at pH 9.0, 298 K.

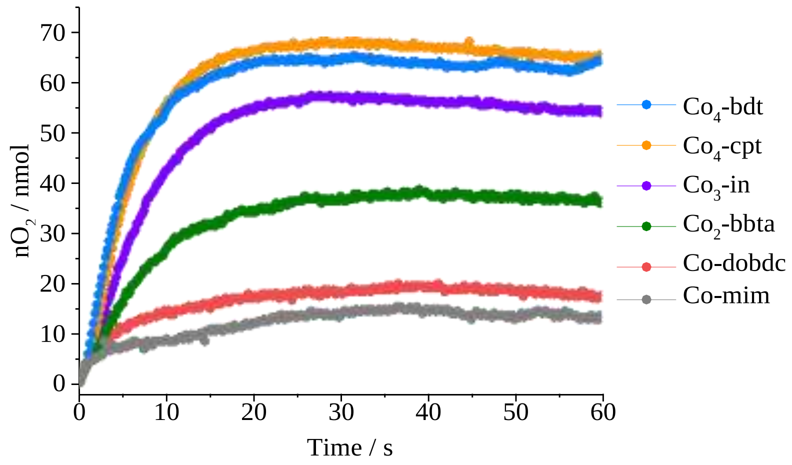

Figure S10. Kinetics of O_2 formation in the oxidation reaction with chemical oxidants $[Ru(bpy)_3]^{3+}$. Conditions: 0.5 nmol catalysts, 15 μM $[Ru(bpy)_3]^{3+}$, 80 mM sodium borate buffer at pH 9.0, 298 K.

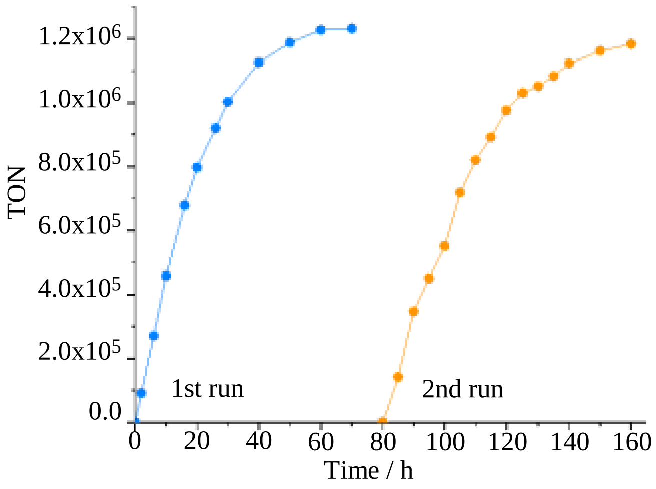

Figure S11. Time-dependent oxygen evolution curves of **Co4-bdt**. Conditions: 0.2 nmol catalysts, 1 M $[Ru(bpy)_3]^{3+}$, 8 M sodium borate buffer at pH 9.0, 298 K. Notably, the TON value of **Co4-bdt** remained 1.2×10^6 after 1 run, indicating that the TON is far greater than one million.

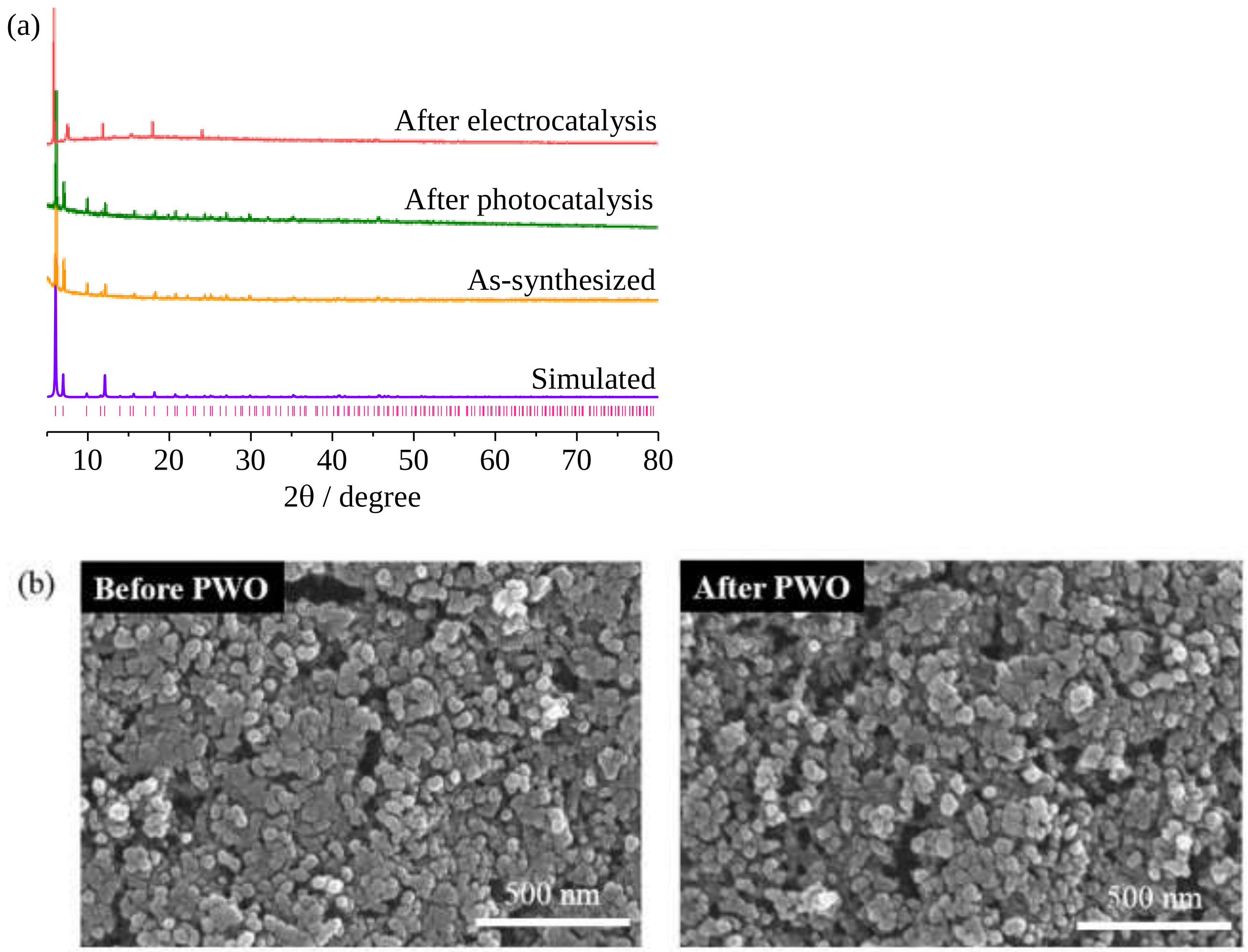

Figure S12. Stability tests of **Co_4-bdt** after photocatalytic measurements in borate buffer (pH =9.0) for 10^4 cycles (a) PXRD patterns and (b) SEM images.

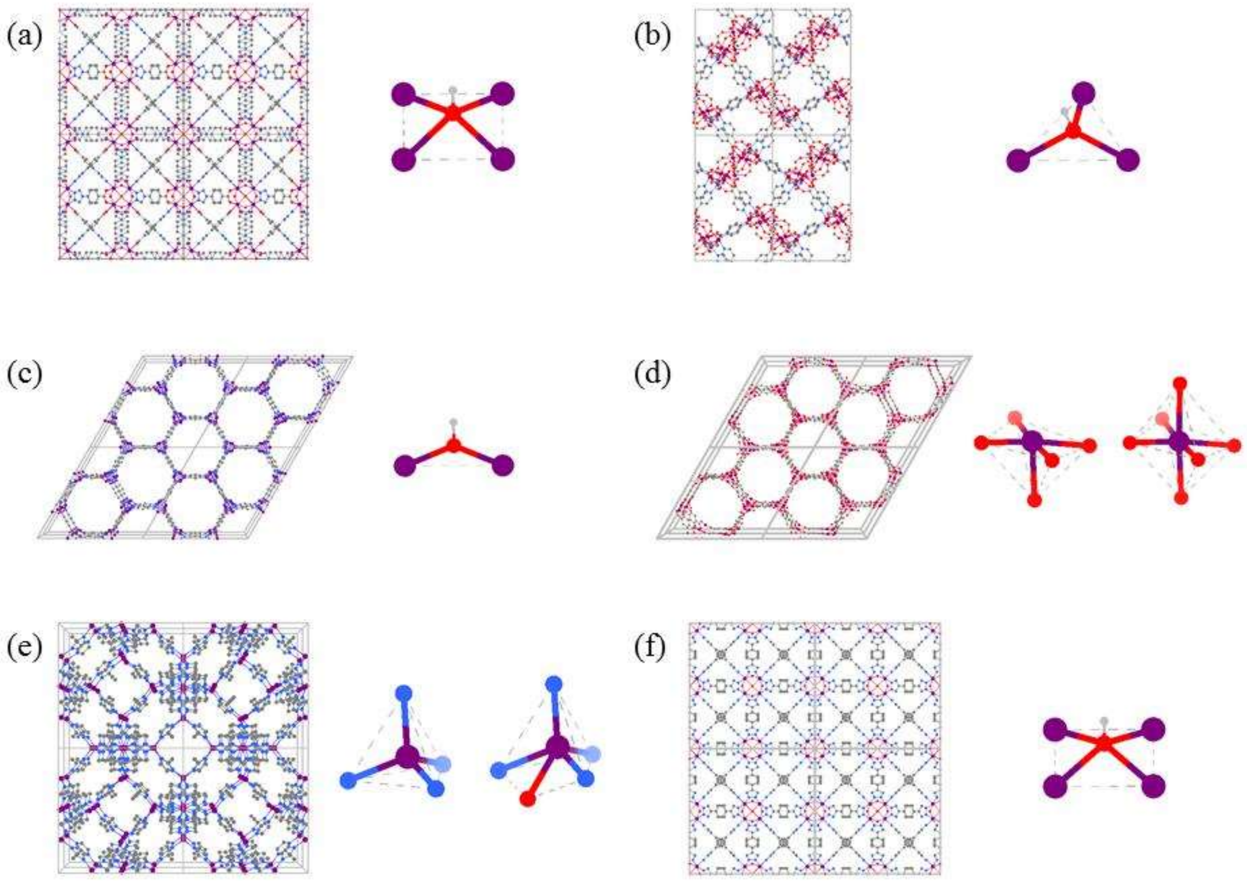

Figure S13. Framework structures and coordination geometries of metal centers (with and without terminal H_2O) of (a) **Co_4-cpt**, (b) **Co_3-in**, (c) **Co_2-bbta**, (d) **Co-dobdc**, (e) **Co-mim** and (f) **Co_4-bdt**. Gray, light gray, red, blue and purple spheres represent C, H, O, N and Co atoms, respectively.

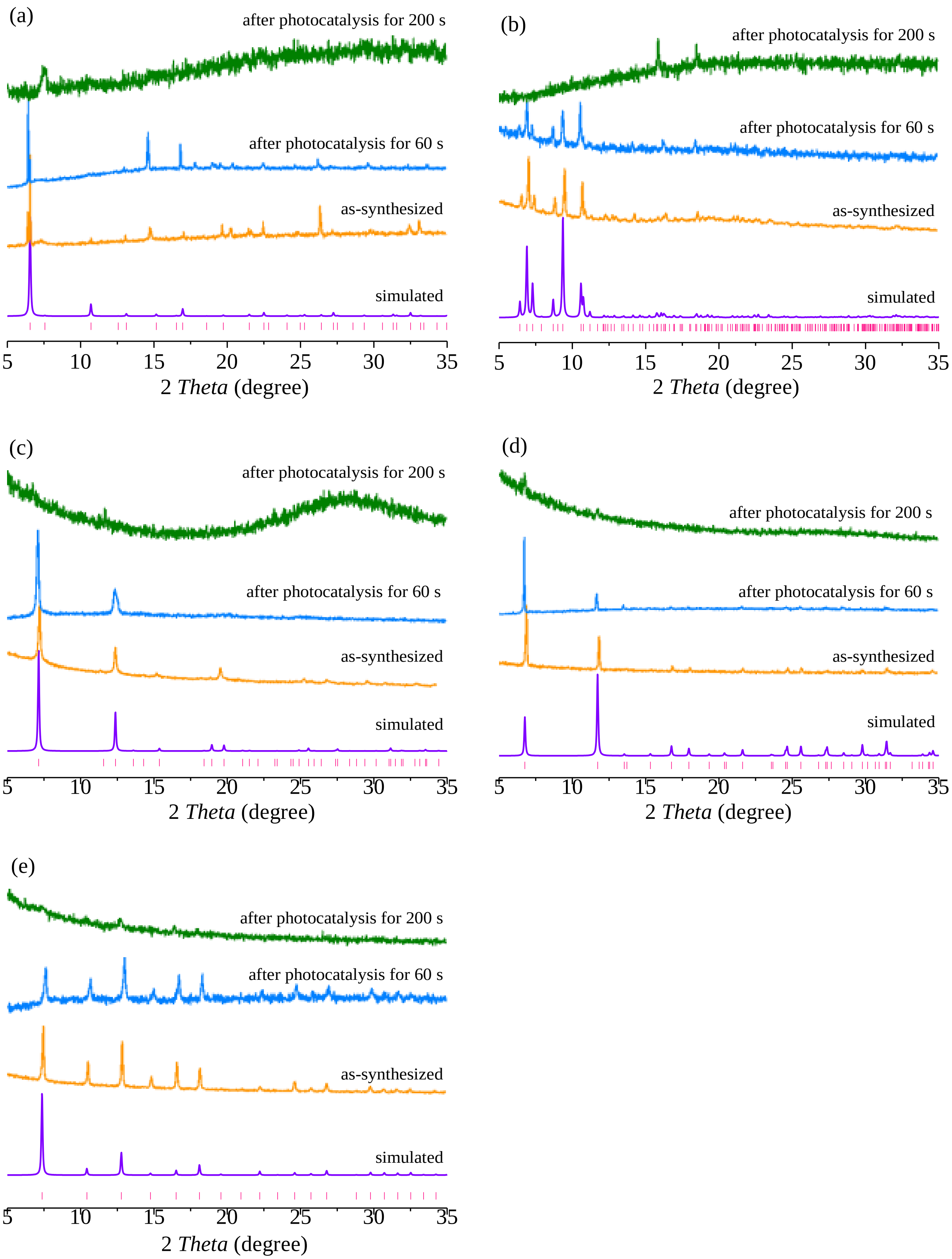

Figure S14. PXRD patterns of (a) **Co4-cpt**, (b) **Co3-in**, (c) **Co2-bbta**, (d) **Co-dobdc** and (e) **Co-mim**.

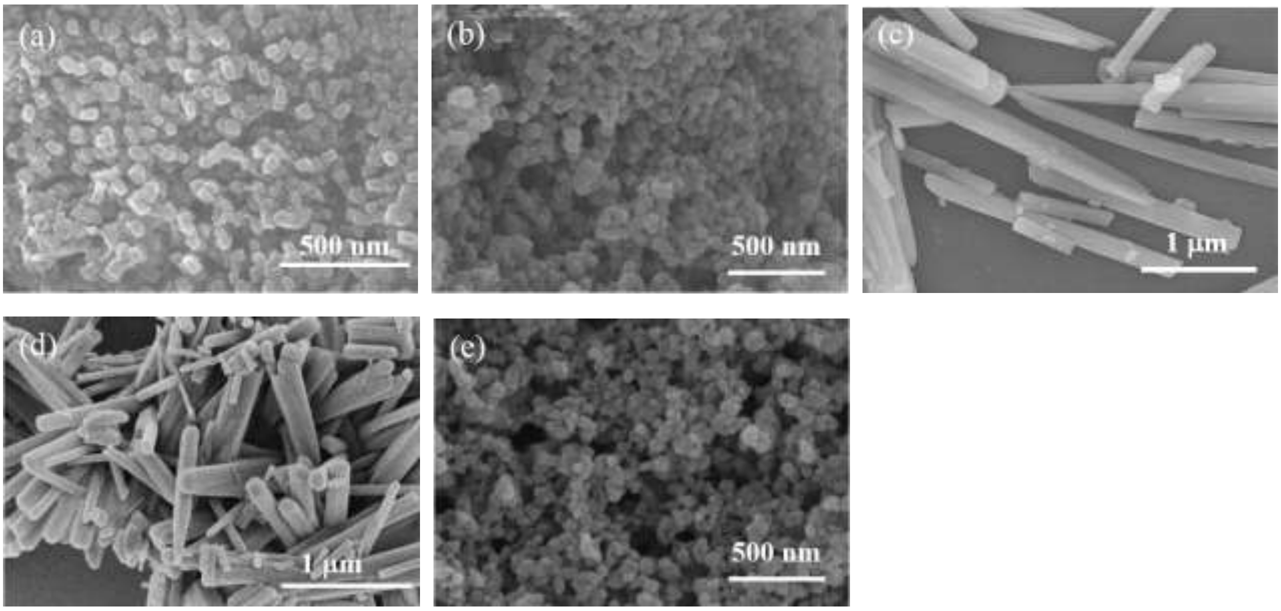

Figure S15. SEM images of (a) **Co4-cpt**, (b) **Co3-in**, (c) **Co2-bbta**, (d) **Co-dobdc**, (e) **Co-mim**.

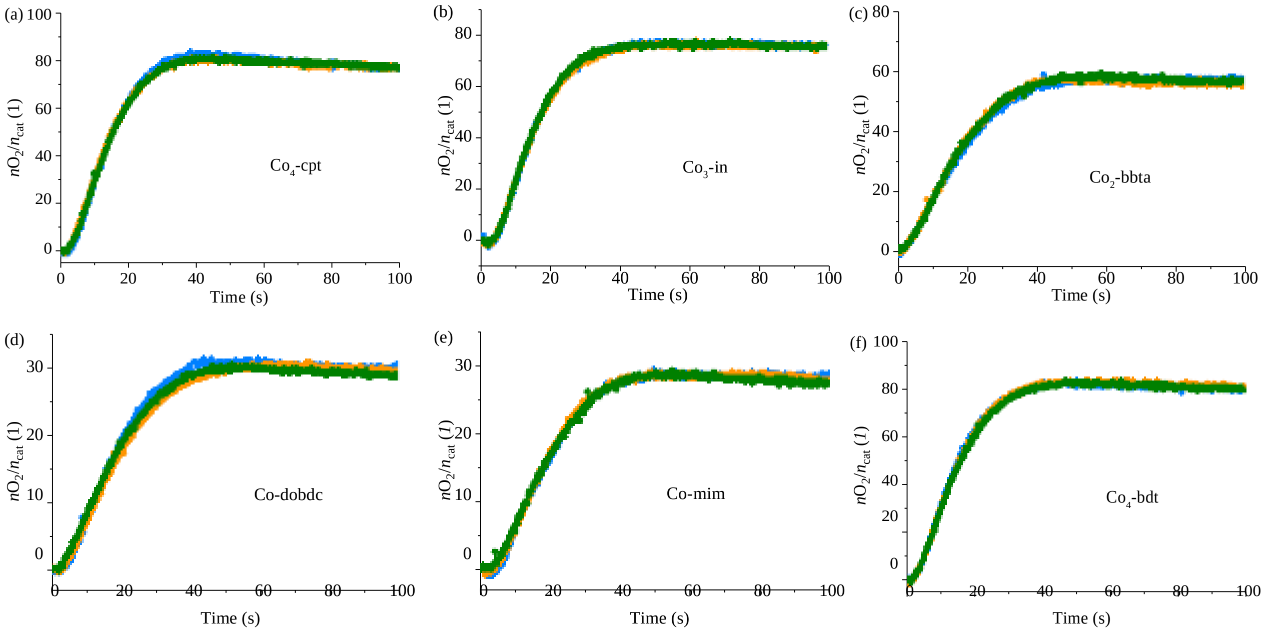

Figure S16. O_2 production profiles of the repeated photocatalytic water oxidation reactions. (a) **Co4-cpt**, (b) **Co3-in**, (c) **Co2-bbta**, (d) **Co-mim** and (e) **Co-dobdc** and (f) **Co4-bdt**.

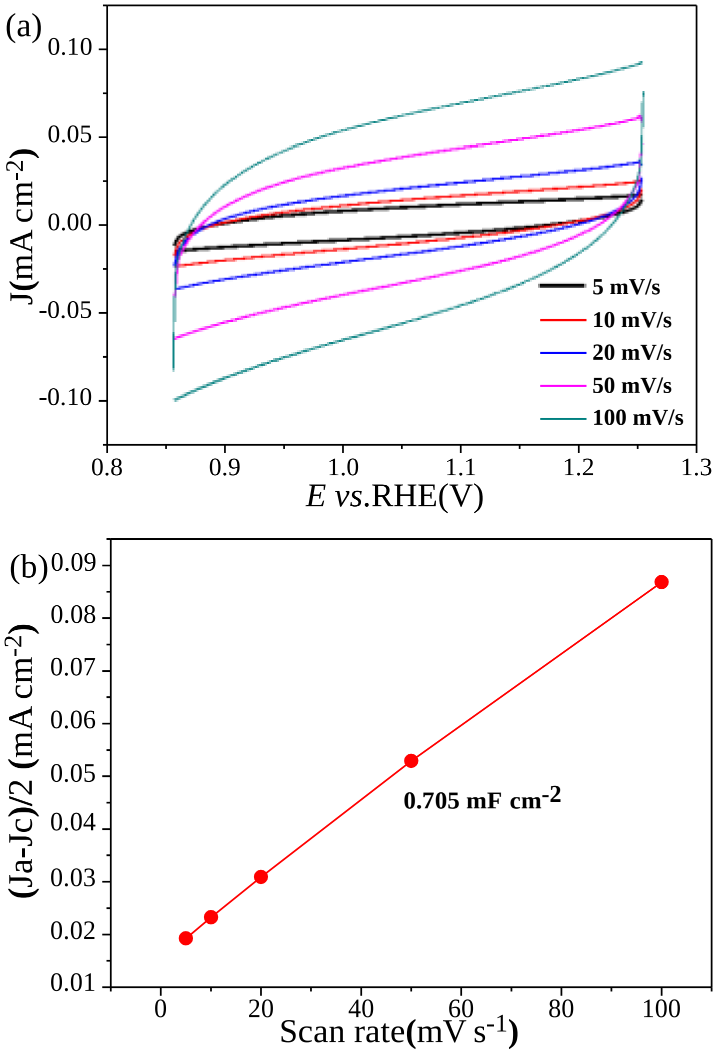

Figure S17. (a) Cyclic voltammetry curves for **Co_4-bdt** at pH = 7; (b) the capacitive current density (Ja/2-Jc/2) as a function of scan rate in the region of 0.85~1.25 V *vs*. RHE.

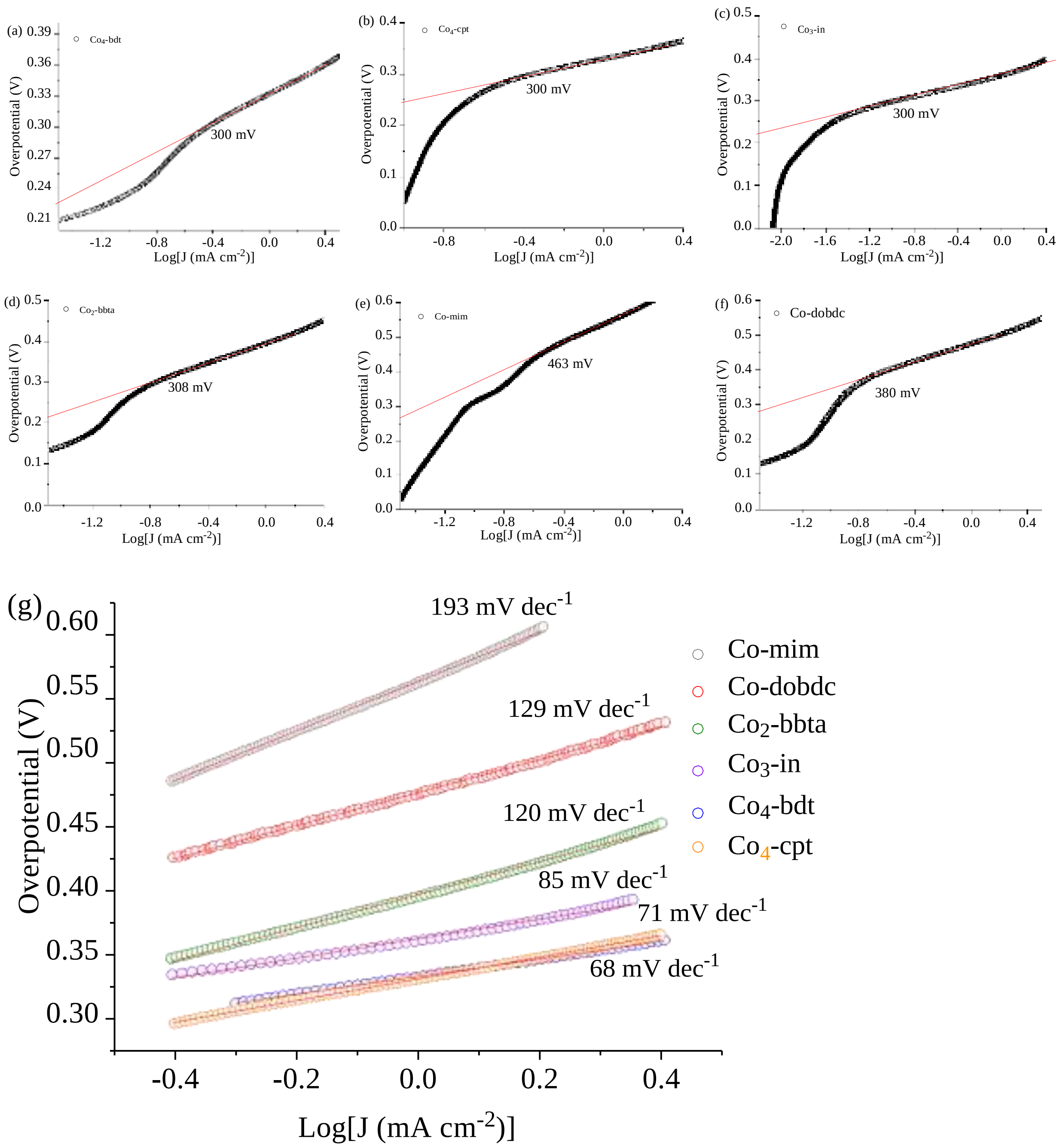

Figure S18. Onset potentials of (a) **Co$_4$-bdt** (b) **Co$_4$-cpt**; (c) **Co$_3$-in**; (d) **Co$_2$-bbta**; (e) **Co-mim** and (f) **Co-dobdc** as well as (g) their Tafel plots at pH = 7.

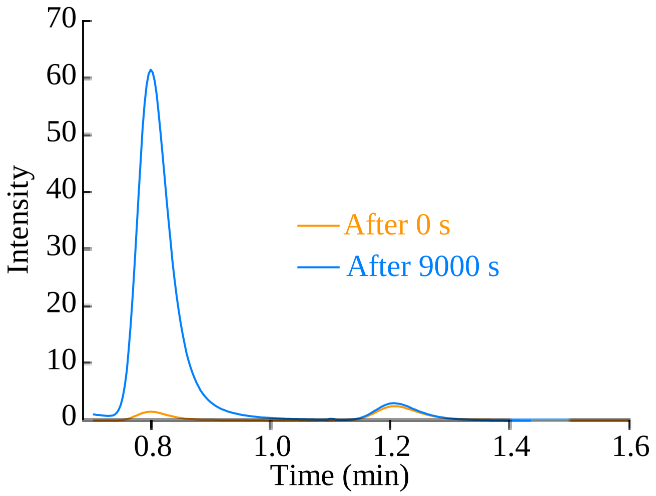

Figure S19. Gas chromatography profiles of O_2 and N_2 in the electrolytic cell using **Co4-bdt** as anode after 0 and 9000 s electrolysis at the current density of 2 mA cm^{-2} in water at pH = 7.

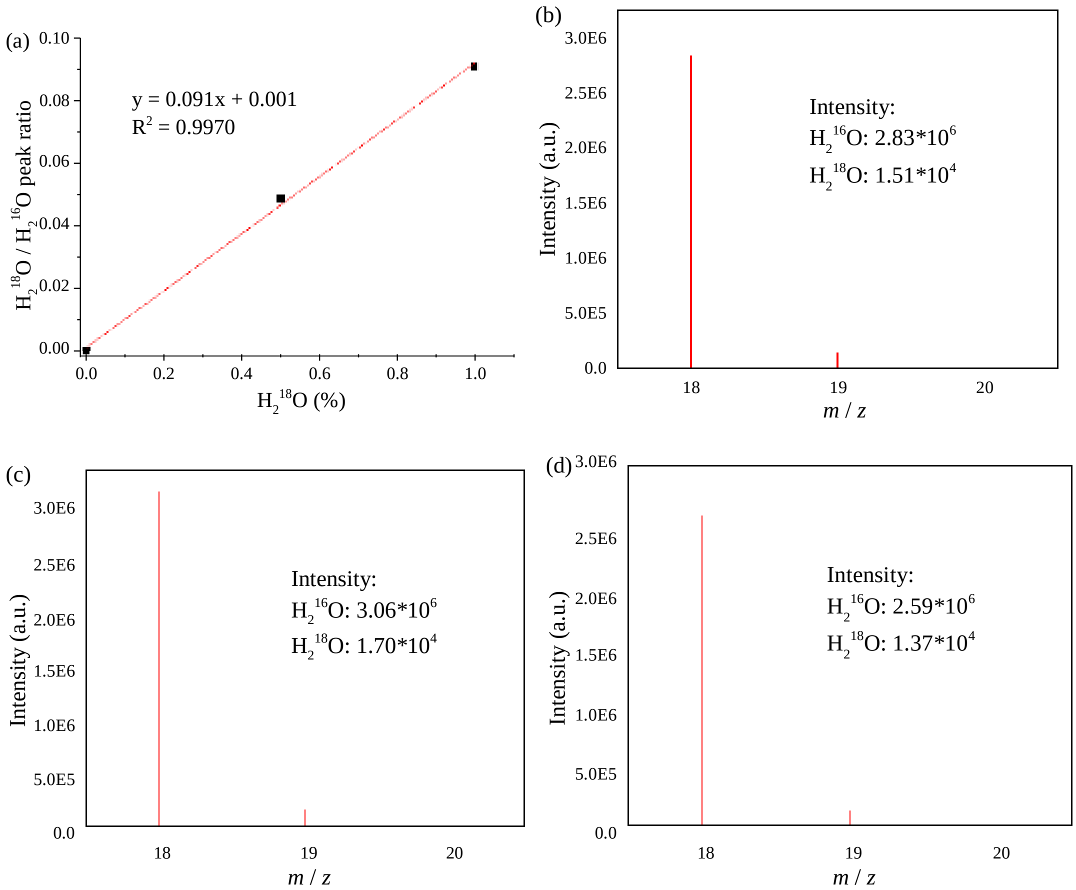

Figure S20. (a) Dependence of the peak ratio of $H_2{}^{18}O/H_2{}^{16}O$ and (b-d) GC-MS profiles of the acidolyzed **Co4-bdt**.

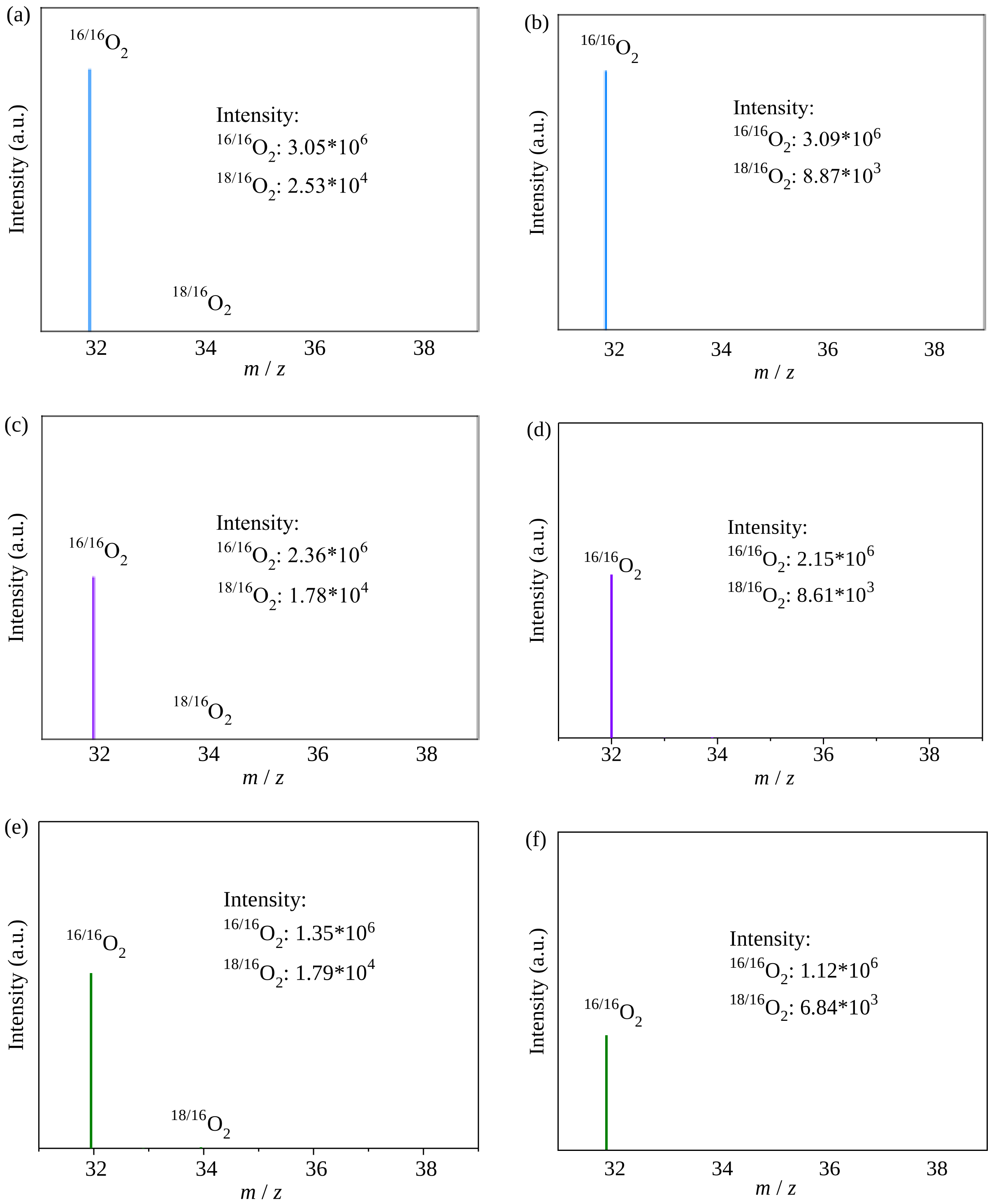

Figure S21. GC-MS profiles of PWO experiments with (a) ^{18}O-labeled or (b) unlabeled **Co_4-bdt**, (c) ^{18}O-labeled or (d) unlabeled **Co_3-in** and (e) ^{18}O-labeled or (f) unlabeled **Co_2-bbta**. Note: The intensity of $^{16}O^{16}O$ (*m*/*z* = 32) is much weaker than that of $^{18}O^{16}O$ (*m*/*z* = 34), which indicated that the ^{18}O-labeled μ_4-OH was exchanged by the water molecule during photodriven water oxidation reaction.

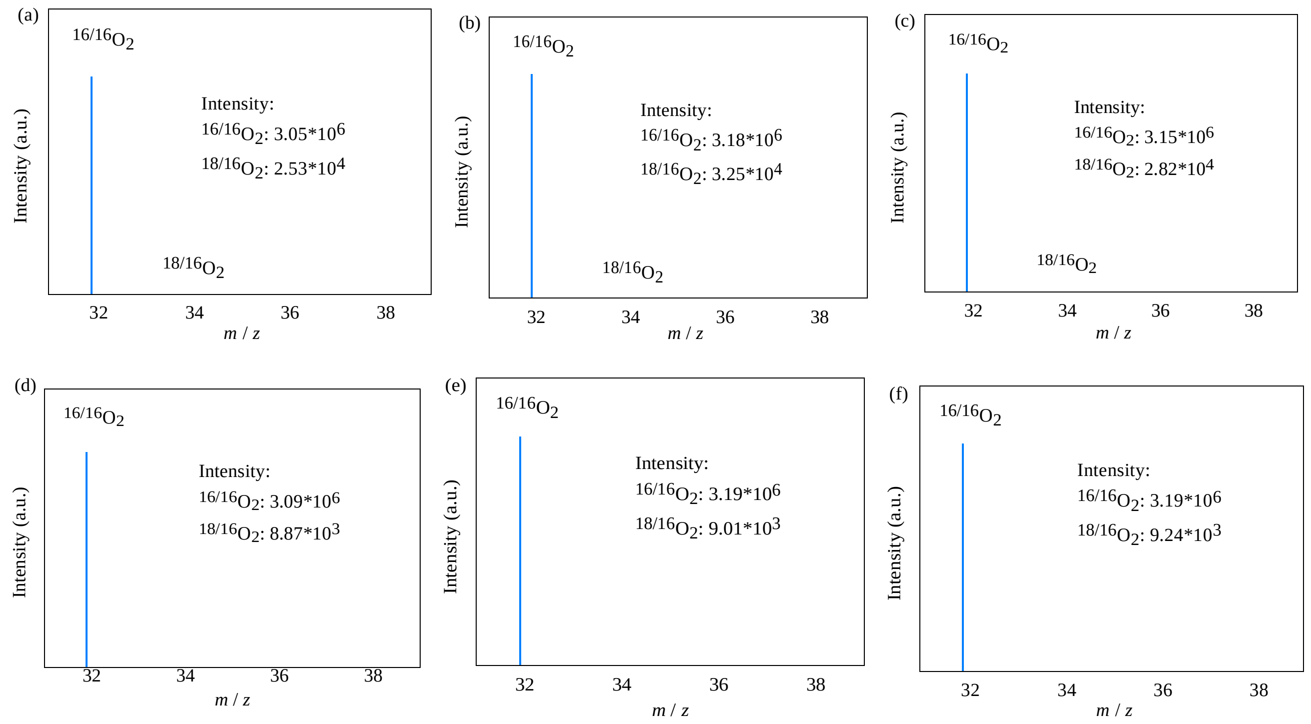

Figure S22. GC-MS profiles of the repeated photocatalytic water oxidation reactions with (a-c) ^{18}O-labeled and (d-f) unlabeled **Co4-bdt**.

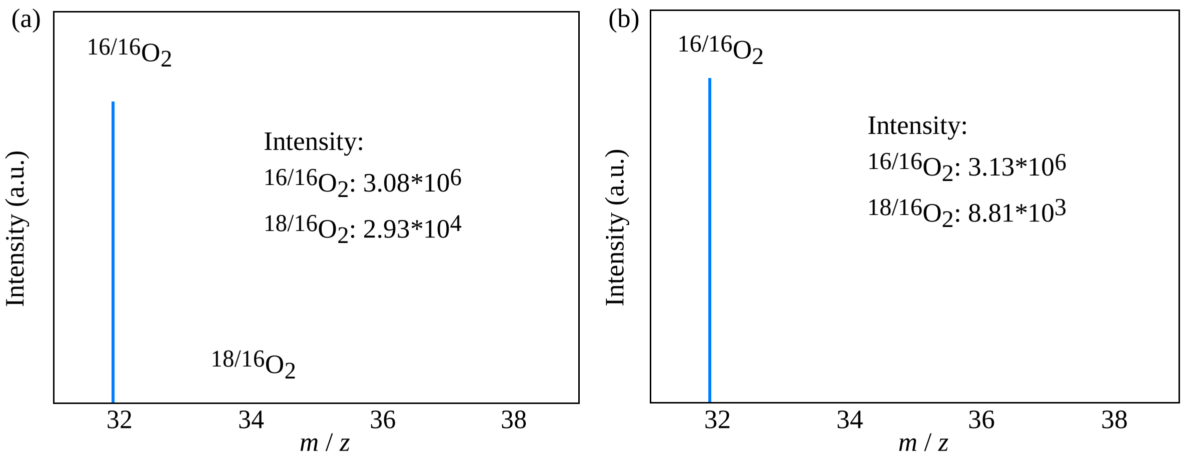

Figure S23. GC-MS profiles of PWO experiments with (a) ^{18}O-labeled **Co4-bdt** (in pH=9 aqueous solution ($H_2{}^{16}O$) for 10 min) and (b) ^{18}O-labeled **Co4-bdt** (after PWO experiment).

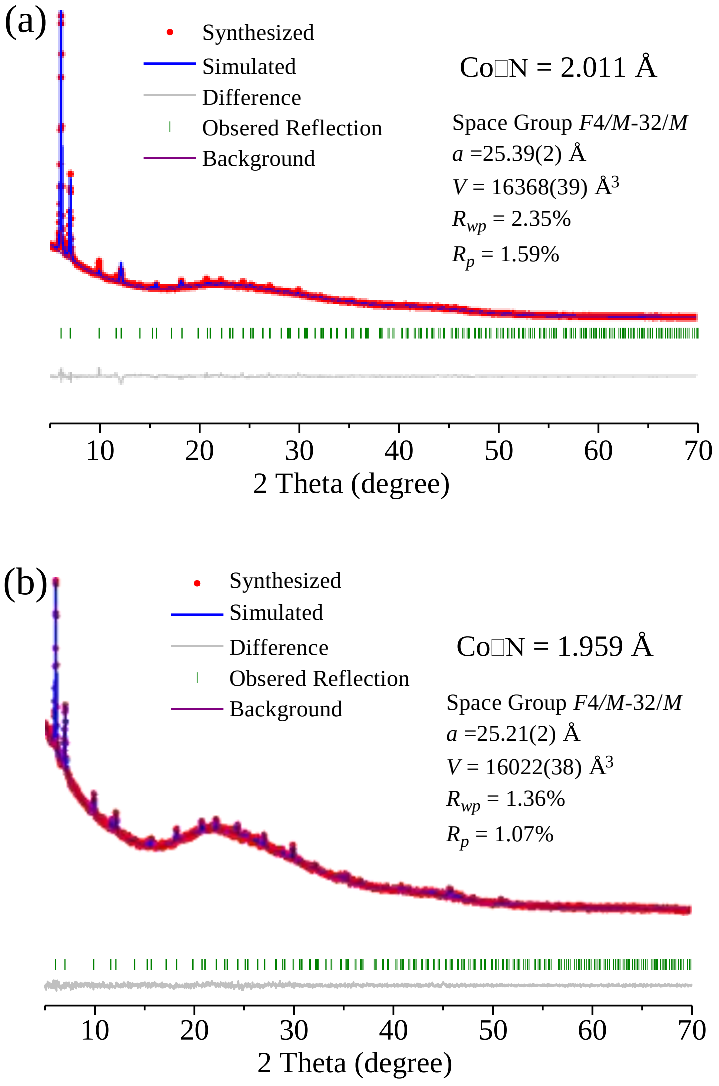

Figure S24. Rietveld refinement plots of **Co$_4$-bdt** (a) before and (b) during PWO experiment.

Table S1. Crystallographic Data for **Co4**-bdt

Complex	**Co4**-bdt
Formula	$C_{60}H_{44}N_{36}O_6Co_8$
Formula weight	1834.73
Temperature (K)	150(2)
Crystal system	Cubic
Space group	*Fm*3*m*
a/Å	25.363(15)
V/Å^3	16315(14)
Z	4
ρ_{calc} /g cm^{-3}	0.750
R_1 [$I > 2\sigma(I)$][a]	0.0501
wR_2 (all data)[b]	0.1553
GOF	1.122

[a] $R_1 = \sum||F_o|-|F_c||/\sum|F_o|$; [b] $wR_2 = \sum[w(F_o^2-F_c^2)^2]/\sum[w(F_o^2)^2]^{1/2}$.

Table S2. Comparison of the photocatalytic activities of **Co_4**-bdt and heterogeneous catalysts.

Meterial	photosensitizer	TOF (s^{-1})	TON	Ref.
**Co_4-bdt** $[Co_8(OH)_6(bdt)_4(Hbdt)_2]$	$[Ru(bpy)_3]SO_4$ (pH = 9.0)	**3.059**	**$>10^6$**	This work
$[Co^{II}(H_2O)_{1.79}]_{1.42}[(Co^{III}_{0.85}Pt^{IV}_{0.15})(CN)_6]$	$[Ru(bpy)_3]SO_4$ (pH = 8.0)	$45.5*10^{-3}$	2.18	*Angew. Chem. Int. Ed.* **2015**, *54*, 5613.
$NiFe_2O_4$	$[Ru(bpy)_3]SO_4$ (pH = 8.0)	$1.24*10^{-3}$	0.75 (1)	*J. Am. Chem. Soc.*, **2012**,*134*, 19572.
Co_3O_4	$[Ru(bpy)_3]SO_4$ (pH = 8.0)	$1.16*10^{-3}$	N/A	
MIL-101(Fe)	$[Ru(bpy)_3]SO_4$ (pH = 10.0)	$41*10^{-3}$	16.8	*Small* **2016**, *12*, 1351
$NiMnO_3$	$[Ru(bpy)_3]SO_4$ (pH = 7.0)	$3.1*10^{-3}$	0.53	*Phys. Chem. Chem. Phys.* **2013**, *15*, 19125.
α-MnO_2	$[Ru(bpy)_3]SO_4$ (pH = 7.0)	$1.16*10^{-3}$	0.59	
$LaCoO_3$	$[Ru(bpy)_3](ClO_4)_2$ (pH = 7.0)	$2.84*10^{-3}$	1.84	*Phys. Chem. Chem. Phys.*, **2012**, *14*, 5753.
$CoWO_4$	$[Ru(bpy)_3](ClO_4)_2$ (pH = 7.0)	$0.756*10^{-3}$	0.52	
Co_3O_4	$[Ru(bpy)_3](ClO_4)_2$ (pH = 7.0)	$2.05*10^{-3}$	1.21	
$La_{0.7}Sr_{0.3}CoO_3$	$[Ru(bpy)_3](ClO_4)_2$ (pH = 7.0)	$1.64*10^{-3}$	1.11	
$NdCoO_3$	$[Ru(bpy)_3](ClO_4)_2$ (pH = 7.0)	$2.40*10^{-3}$	NA	
$YCoO_3$	$[Ru(bpy)_3](ClO_4)_2$ (pH = 7.0)	$0.70*10^{-3}$	NA	
Co-ZIF-67	$[Ru(bpy)_3](ClO_4)_2$	$35*10^{-3}$	52.5	*New J. Chem.*, **2016**, *40*, 3032
MIL-101(Fe)	$[Ru(bpy)_3](ClO_4)_2$ (pH = 10.0)	$100*10^{-3}$	27.3	*Small* **2016**, *12*, 1351
NiO/CoO/Fe_2O_3	$[Ru(bpy)_3]Cl_2$ (pH = 8.5)	$1.16*10^{-3}$	1.89	*Dalton Trans.* **2015**, *44*, 15628.
CoNCN	$[Ru(bpy)_3]Cl_2$ (pH = 9.0)	$29.4*10^{-3}$	NA	*J. Mater. Chem. A*, **2015**, *3*, 5072
CoPOM/MIL-101(Cr)	$[Ru(bpy)_3]Cl_2$	$7.1*10^{-3}$	8.52	*Applied Catalysis A: General*, **2016**, *521*, 83
Mn_2O_3 (bixbyite)	$[Ru(bpy)_3]Cl_2$ (pH = 8.0)	$0.37*10^{-3}$	NA	*J. Am. Chem. Soc.* **2013**, *135*, 3494.
Mn_3O_4 (hausmannite)	$[Ru(bpy)_3]Cl_2$ (pH = 8.0)	$0.16*10^{-3}$	NA	
λ-MnO_2 (spinel)	$[Ru(bpy)_3]Cl_2$ (pH = 8.0)	$0.055*10^{-3}$	NA	

Table S3. Comparison of the photocatalytic activities of **Co_4**-bdt and homogeneous catalysts. The parameters better than that of **Co_4**-bdt are highlighted in blue.

Meterial	photosensitizer	TOF (s^{-1})	TON	Ref.
**Co_8**	$[Ru(bpy)_3]SO_4$	**3.059**	**>10^6**	This work
PSII	$[Ru(bpy)_3]Cl_2$	10^7	500	*Biochem. J.* **1980**, *188*, 351
$[(A\text{-}\alpha\text{-}SiW_9O_{34})_2Co_8(OH)_6(H_2O)_2(CO_3)_3]^{16-}$	$[Ru(bpy)_3]Cl_2$	10	1436	*ChemSusChem* **2015**, *8,* 2630
$[Co^{II}_4(hmp)_4(\mu\text{-}OAc)_2(\mu_2\text{-}OAc)_2(H_2O)_2]$	$[Ru(bpy)_3]Cl_2$	7	40	*J. Am. Chem. Soc.*, **2013**, *135*, 18734.
$[Co^{III}_4O_4(OAc)_4(py)_4]$	$[Ru(bpy)_3]Cl_2$	0.02	40	*J. Am. Chem .Soc.*, **2011**, *133,* 11446.
$[Co^{III}_4O_4(OAc)_4(p\text{-}C_5H_4X)_4]$	$[Ru(bpy)_3]Cl_2$	0.07	140	*J. Am. Chem. Soc.*, **2012**, *134*, 11104.
$[Co_3Ln(hmp)_4(OAc)_5H_2O]$ (Ln = Er, Tm, Yb)	$[Ru(bpy)_3]Cl_2$	9	160-200	*J. Am. Chem. Soc.*, **2015**, *137*, 11076.
$[Co(qpy)(OH_2)_2]^{2+}$	$[Ru(bpy)_3]Cl_2$	4	335	*Energy Environ. Sci.* **2012,** *5,* 7903.
$Na_{30}K_2[_4(GeW_9O_{34})_4]51H_2O$	$[Ru(bpy)_3]Cl_2$	0.1	38.75	*J. Am. Chem. Soc.* **2014**, *136*, 5359
$Na_{50}[Ni_{25}(H_2O)_2OH)_{18}(CO_3)_2(PO_4)_6(SiW_9O_{34})_6]$	$[Ru(bpy)_3]Cl_2$	0.34	204.5	*J. Am. Chem. Soc.* **2015**, *137,* 5486.
CoTPPS	$[Ru(bpy)_3](NO_3)_2$	0.17	121.8	*Chem. Commun.* **2013,** *49*, 6325
Salen Co(II)	$[Ru(bpy)_3](ClO_4)_2$	6.4	854	*Chem. Commun.* **2014**, *50*, 2167
$Na_{10}[Co_4(H_2O)_2(PW_9O_{34})_2]27H_2O$	$[Ru(bpy)_3](ClO_4)_2$	5	75	*Science*, **2010**, *328*, 342.

Table S4. Summary of the results of the photocatalytic water oxidation.

Meterial	TOF^{1st} (s^{-1})	$\Phi_{O2}{}^{1st}$ (%)	TOF^{2nd} (s^{-1})	$\Phi_{O2}{}^{2nd}$ (%)
**Co_4-bdt**	3.054	1.61	3.031	1.60
**Co_4-cpt**	3.018	1.59	1.498	0.79
**Co_3-in**	2.369	1.24	0.483	0.25
**Co_2-bbta**<br>MAF-X27-OH	1.745	0.92	0.694	0.37
**Co-dobdc**<br>Co-MOF-74	0.766	0.40	0.380	0.20
**Co-mim**<br>ZIF-67	0.576	0.31	0.366	0.19

All photochemical experiments in this work were carried out under exposure to monochromatic light beam with an excitation wavelength of 450 nm. The apparent quantum yield of O_2 (Φ_{O2}):

Φ_{O2} = [2×(number of the produced molecule) / (number of photons)] ×100%

Photon flux = (I×S) / (h×c / λ) = 1.9×10^{-7} mol/s.

Light intensity (I) = 100 mW/cm^2. Area (S) = 0.50 cm^2. λ = 450 nm.

h is Planck constant = 6.62606957×10^{-34} J s , c is velocity of light = 3×10^{8} m/s.

Note that the apparent quantum yield calculation does not consider the catalyst amount and many other aspects (such as the transparency of the reaction container), meaning that only the experiments carried out with the same setting are suitable for comparison of the apparent quantum yields.

Table S5. Summary of the results of the repeated photodriven water oxidation reaction.

MOFs	**Co_4-bdt**	**Co_4-cpt**	**Co_3-in**	**Co_2-bbta**	**Co-dobdc**	**Co-mim**
I	3.033	2.960	2.294	1.721	0.746	0.586
II	3.033	3.021	2.410	1.780	0.749	0.585
III	3.097	3.021	2.405	1.735	0.803	0.556
Average	3.054	3.018	2.369	1.743	0.766	0.576
	3.054±0.030	3.018±0.046	2.369±0.054	1.745±0.025	0.766±0.026	0.576±0.014

Table S6. The obtained gas chromatography peak areas of O_2/N_2 in the electrolytic cell are:

	O_2	N_2	O_2/N_2 Ratio
Air	1462.3	4352.1	0.336
After 0 s	8.967	21.20	0.423
After 9000 s	209.38	21.63	9.68

The GC peak area (denoted as *A*) has a relationship: *A* (oxygen produced) = *A* (oxygen 9000 s) – *A* (oxygen 0 s) – *A* (oxygen leak from air) in which *A* (oxygen leak from air) = *A* (nitrogen leak from air) × (O_2/N_2 Ratio) = (*A* (nitrogen 9000 s) – *A* (nitrogen 0 s)) × 0.336 = (21.63– 21.20) × 0.336 = 0.144.

Therefore, *A* (oxygen produced) = *A* (oxygen 9000 s) – *A* (oxygen 0 s) – *A* (oxygen leak from air) = 209.38– 8.967 – 0.144 = 200.27.

Giving n_{O2} = O_2 concentration(%) × head space volume / (22.4 × 298 / 273) = (200.27 + 0.968)/5772.14 × 32.0 / 1000 / 24.45 mol = 4.563 × 10^{-5} mol = 4.563 μmol. Because n_{cat} = mass loading (mg cm^{-2}) × electrode area (cm^2) / molecular weight / 1000 = 8 × 0.22 × 0.5 / 2213 / 1000 =3.976 × 10^{-7} mol, TOF = 4.563 × 10^{-5} / 3.976 × 10^{-7} / 18000 = 0.00638 s^{-1}.

Considering all Co ions in **Co_4-bdt** (2213 g mol^{-1}) as catalytic centers, M = 2213/8 = 276.62 g mol^{-1}. $TOF_{theoretical}$ = 2.0 / (4 × 96485 × 0.22 / 276.62) = 0.00652 s^{-1} for **Co_4-bdt** at 2.0 mA cm^{-2}.

F_{OER} = 0.00638 s^{-1}/0.00652 s^{-1} × 100% = 97.8%

Considering there is small amount of O_2 dissolved in the solution which have been omitted (can be hardly measured accurately), the Faraday efficiency of **Co_4-bdt** for OER can be considered as near/virtually 100%.

Table S7. Comparison of the OER activities of **Co_4-bdt** and benchmarked catalysts working at pH = 7.

Catalyst	Onset potential (V vs. RHE)	Overpotential at 2 mA cm^{-2}(mV vs. RHE)	Tafel slope (mV dec^{-1})	Mass loading (mg cm^{-2})	Substrate	Binder	Ref
Co_4-bdt	1.54	352	71	0.22	GC	Nafion	**This work**
Co_4-cpt	1.54	355	68	0.22	GC	Nafion	
Co_3-in	1.54	385	85	0.22	GC	Nafion	
Co_2-bbta	1.548	489	120	0.22	GC	Nafion	
Co-dobdc	1.620	544	129	0.22	GC	Nafion	
Co-mim	1.703	638	193	0.22	GC	Nafion	
Fe_3-Co_2	1.623	463	170	0.2	GC	Nafion	*J. Am. Chem. Soc.* **2017**, *139*, 1778
MOF-74-Co	1.641	545	127	0.2	GC	Nafion	
ZIF-67	1.688	639	192	0.2	GC	Nafion	
MAF-X27-OH	1.548	489	127	0.2	GC	Nafion	*J. Am. Chem. Soc.*, **2016**, *138*, 8336.
Composite of cobalt(II) 5,10,15,20-tetraethynyl-porphyrin complex and multiwall carbon nanotube	NA	620 (at 0.2 mA cm^{-2})	150	0.1	GC	Nafion	*Chem. Mater.* **2015**, *27*, 4586
$Co(OH)_2$	1.69	710 (at 1 mA cm^{-2})	400	NA	GC	Nafion	*Nanoscale* **2013**, *5*, 6826
Fe based film	NA	550	NA	2.78	GC	Nafion	*Angew. Chem. Int. Ed.* **2015**, *54*, 4870
NiO_x	NA	760	NA	NA	ITO glass	Nafion	*Adv. Energy Mater.* **2014**, *4*, 1301929
Mn oxide film	NA	620	80	NA	ITO glass	Nafion	*Energy Environ. Sci.* **2012**, 5, 7081
$K_{2x}Co_{(2-x)}[Fe(CN)_6]$	NA	470 (at 0.1 mA cm^{-2})	NA	NA	ITO glass	Nafion	*J. Am. Chem. Soc.* **2013**, *135*, 13270.
$Co_3(PO_4)_2$	NA	460	NA	NA	ITO glass	Null	*Science* **2008**, *321*, 1072
Fe based film	1.70	504	47	12.3 nmol	ITO glass	Nafion	*ACS Appl. Mater.*

				cm^{-2}			*Interfaces*, *2015*, *7*, *21852*.
$Co(OH)_2$	1.59	650	NA	NA	FTO glass	Nafion	*Nanoscale* **2014**, *6*, 3376
CoNi LDHs	1.623	490 (at 1 mA cm^{-2})	230	NA	FTO glass	Nafion	*Phys. Chem. Chem. Phys.* **2013**, *15*, 7363
Mn_5O_8	NA	513	154	NA	FTO glass	Nafion	*ACS Catal.* **2015**, *5*, 4624
$Au/Co(OH)_2$	NA	650	370	NA	FTO glass	Nafion	*Nanoscale* **2013**, *5*, 6826
Co-ZIF-9	1.6	690 (at 0.6 mA cm^{-2})	193	0.3	FTO glass	Nafion	*Nanoscale*, **2014**, *6*, 9930
$Co_{0.66}Cu_{0.34}(CO_3)_{0.5}(OH)$	1.75	800	NA	NA	FTO glass	Nafion	*Nanoscale* **2014**, *6*, 3376
$ZnCo_2O_4$	NA	540	85	NA	Pt-Ti alloy coated glass	Nafion	*J. Phys. Chem. Lett.* **2014**, *5*, 2370
$LiMnP_2O_7$	NA	600 (at 0.2 mA cm^{-2})	NA	0.25	Al foil	Nafion	*J. Am. Chem. Soc.* **2014**, *136*, 4201

Table S8. Summary of the $H_2{}^{16}O$ and $H_2{}^{18}O$ peak intensity of the acidolyzed **Co_4-bdt**.

Run	$H_2{}^{16}O$	$H_2{}^{18}O$	$H_2{}^{18}O$ / $H_2{}^{16}O$
I	2.83×10^6	1.51×10^4	5.33×10^{-3}
II	3.06×10^6	1.70×10^4	5.55×10^{-3}
III	2.59×10^6	1.37×10^4	5.30×10^{-3}
Average	2.83×10^6	1.53×10^4	5.40×10^{-3}
	$2.83 \pm 0.19 \times 10^6$	$1.53 \pm 0.13 \times 10^4$	$5.40 \pm 0.11 \times 10^{-3}$

100 μmol of **Co_4-bdt** was dissolved in 1mL concentrated sulfuric acid. The pH value of the solution was carefully adjusted to neutral by NaOH and the solution was diluted with water to 10 mL (556 mmol H_2O). Theoretically, the extent of ^{18}O was calculated as 100*6 μmol / 556 mmol = 0.108%. As shown in Figure S25 and Table S9, the peak ratio of $H_2{}^{18}O/H_2{}^{16}O = 5.40*10^{-3}$. Based on the standard curve, $H_2{}^{18}O\% = (5.40*10^{-3} – 0.001) / 0.091 = 0.0484\%$. Therefore, the extent of ^{18}O catalyst incorporation was calculated as 0.0476% / 0.108% = 44.8%.

Table S9. Summary of the $^{18/16}O_2$ peak intensity of the photodriven water oxidation reaction.

Run	^{18}O-labeled	Unlabeled
I	2.53×10^4	8.87×10^3
II	3.25×10^4	9.01×10^3
III	2.82×10^4	9.24×10^3
Average	2.87×10^4	9.04×10^3
	$2.87 \pm 0.29 \times 10^4$	$9.04 \pm 0.15 \times 10^3$

References

(1) Huang, Z.; Luo, Z.; Geletii, Y. V.; Vickers, J. W.; Yin, Q.; Wu, D.; Hou, Y.; Ding, Y.; Song, J.; Musaev, D. G.; Hill, C. L.; Lian, T., Efficient Light-Driven Carbon-Free Cobalt-Based Molecular Catalyst for Water Oxidation. *J. Am. Chem. Soc.* **2011,** *133*, 2068-2071.

(2) Lu, X.-F.; Liao, P.-Q.; Wang, J.-W.; Wu, J.-X.; Chen, X.-W.; He, C.-T.; Zhang, J.-P.; Li, G.-R.; Chen, X.-M., An Alkaline-Stable, Metal Hydroxide Mimicking Metal–Organic Framework for Efficient Electrocatalytic Oxygen Evolution. *J. Am. Chem. Soc.* **2016,** *138*, 8336-8339.
